# Supplementary material for: Alkyne-tagged SERS nanoprobe for understanding Cu+ and Cu2+ conversion in cuproptosis processes
Source: Nat Commun. 2024 Apr 15;15:3246. doi: 10.1038/s41467-024-47549-1 (PMC11018805; doi:10.1038/s41467-024-47549-1)
Supplement: Supplementary file 1 — Supporting Information [file 41467_2024_47549_MOESM1_ESM.pdf]

Supplementary Information

Alkyne-tagged SERS nanoprobe for understanding Cu^{+} and Cu^{2+} conversion in cuproptosis processes

Sihan Zhang[1], Yuxiao Mei[1], Jiaqi Liu[1], Zhichao Liu[1]*, and Yang Tian[1]*

[1] State Key Laboratory of Molecular & Process Engineering, School of Chemistry and Molecular Engineering, East China Normal University, Dongchuan Road 500, Shanghai 200241, China

*E-mail: ytian@chem.ecnu.edu.cn (Y. Tian); zcliu@chem.ecnu.edu.cn (Z. Liu)

Contents

1. **Supplementary Methods**
2. **Supplementary Discussion**
3. **Supplementary Figures (Figs. 1-30)**
4. **Supplementary Tables (Tables 1-11)**
5. **Supplementary References**

1. Supplementary Methods

Synthesis of methyl 4-((4-(chloromethyl) phenyl) buta-1,3-diyn-1-yl) benzoate (2).

A mixture of CuCl (0.50 g, 5.00 mmol) and TMEDA (1.50 mL, 10.00 mmol) in 25.00 mL of acetone was stirred at 25 °C for 10 min with the constant bubbling of air. Then, a mixture of (4-ethynylphenyl) methanol (1.32 g, 10.00 mmol) and 4-ethynylbenzoic acid methyl ester (4.80 g, 30.00 mmol) in 15.00 ml dichloromethane was added to the above solution, and the mixture was stirred at 25 °C with air for 2 h. After the reaction was completed, the mixture was filtered to collect the filtrate, and the solvent was removed in a vacuum. The crude product was purified by column chromatography (silica gel, methanol/dichloromethane=1/10) to give a yellow solid (0.99 g, 34.14% yield). Then a mixture of the above product (0.99 g, 3.40 mmol) and $SOCl_2$ (0.36 mL, 5.00 mmol) in 10.00 mL chloroform was stirred at 60 °C for 12 h under a nitrogen atmosphere. After the reaction was completed, the solvent was removed in a vacuum and the residue was boiled with 50 mL dichloromethane to remove residual $SOCl_2$. After drying in a vacuum, a pale-yellow solid Compound 2 was obtained (0.99 g, 94.16% yield). ^{1}H NMR (500 MHz, $CDCl_3$) δ (ppm): 8.01-8.00 (d, J = 8.0 Hz, 2H), 7.59-7.57 (d, J = 8.0 Hz, 2H), 7.53-7.52 (d, J = 8.0 Hz, 2H), 7.38-7.36 (d, J = 8.0 Hz, 2H), 4.58 (s, 2H), 3.92 (s, 3H). ^{13}C NMR (500 MHz, $CDCl_3$) δ (ppm): 166.30, 138.78, 132.90, 132.42, 130.39, 129.57, 128.71, 126.35, 121.59, 82.40, 80.88, 76.57, 74.28, 52.35 and 45.60. HR-MS (ESI) m/z for $C_{19}H_{13}ClNaO_2$ $[M+Na]^+$ calcd. 331.0496, found: 331.0504.

Synthesis of methyl 4-((4-((bis(2-((2-(ethylthio) ethyl) thio) ethyl) amino) methyl) phenyl) buta-1,3-diyn-1-yl) benzoate (3).

A mixture of Compound 1 (0.81 g, 2.60 mmol),[1] Compound 2 (0.99 g, 3.20 mmol) and K_2CO_3 (1.08 g, 7.80 mmol) in 20 mL acetonitrile was stirred at 80 °C under nitrogen atmosphere for 12 h. After the reaction was completed, the mixture was filtered to collect the filtrate, and the solvent was removed in a vacuum. The residue was purified by column chromatography (silica gel, petroleum ether/dichloromethane=1/3) to give a reddish-brown viscous liquid Compound 3 (1.05 g, 69.04% yield). ^{1}H NMR (500 MHz, $CDCl_3$) δ (ppm): 8.03-8.01 (d, J = 8.5 Hz, 2H), 7.60-7.59 (d, J = 8.5 Hz, 2H), 7.52-7.50 (d, J = 7.5 Hz, 2H), 7.38-7.36 (d, J = 7.5 Hz, 2H), 3.94 (s, 3H), 3.67 (s, 2H), 2.76-2.65 (m, 16H), 2.59-2.54 (m, 4H), 1.29-1.26 (t, 6H). ^{13}C NMR (500 MHz, $CDCl_3$) δ (ppm): 166.32, 141.26, 132.60, 132.37, 130.25, 129.54, 128.79, 126.52, 120.18, 83.09, 83.05, 80.45, 80.44, 58.42, 53.97, 52.32, 32.42, 31.78, 30.15, 26.08 and 14.82. HR-MS (ESI) m/z for $C_{31}H_{39}NNaO_2S_4$ $[M+Na]^+$ calcd. 608.1756, found: 608.1776.

Synthesis of 4-((4-((bis(2-((2-(ethylthio) ethyl) thio) ethyl) amino) methyl) phenyl) buta-1,3-diyn-1-yl)-N-(prop-2-yn-1-yl) benzamide (Cu^1R_5).

A mixture of propargylamine (0.11 g, 2.00 mmol) and trimethylaluminum (2.00 mL, 1 M in n-hexane) was stirred at 0 °C under nitrogen atmosphere for 10 min, and then a solution of Compound 3 (1.05 g, 1.80 mmol) in 20.00 mL n-hexane was added dropwise. Next, the mixture was warmed to 50 °C and stirred for 3 h. After the reaction was completed, the solvent was removed in a vacuum, and the residue was purified by column chromatography (silica gel, methanol/dichloromethane=1/50) to obtain a brown viscous liquid as ligand Cu^1R_5 (0.74 g, 67.59% yield). ^{1}H NMR (500 MHz, $CDCl_3$) δ (ppm): 7.79-7.77 (d, *J* = 8.0 Hz, 2H), 7.60-7.59 (d, *J* = 8.0 Hz, 2H), 7.51-7.50 (d, *J* = 7.5 Hz, 2H), 7.38-7.36 (d, *J* = 7.5 Hz, 2H), 6.47-6.45 (t, 1H), 4.27-4.26 (m, 2H), 3.68 (s, 2H), 2.76-2.65 (m, 16H), 2.59-2.54 (m, 4H), 2.31-2.30 (t, 1H), 1.29-1.26 (t, 6H). ^{13}C NMR (500 MHz, $CDCl_3$) δ (ppm): 166.15, 147.78, 133.80, 132.65, 132.61, 128.83, 127.15, 125.49, 120.51, 79.28, 79.25, 77.26, 76.46, 76.42, 72.08, 58.41, 53.97, 32.42, 31.78, 30.10, 29.70, 26.09 and 14.83. HR-MS (ESI) m/z for $C_{33}H_{40}N_2NaOS_4$ $[M+Na]^+$ calcd. 608.1756, found: 631.1927.

Synthesis of N, N-bis(pyridin-2-ylmethyl) prop-2-yn-1-amine (4).

A mixture of propargyl bromide (1.42 g, 12.00 mmol), bis (pyridin-2-ylmethyl) amine (1.99 g, 10.00 mmol), KI (0.10 g, 0.60 mmol) and K_2CO_3 (1.70 g, 12.00 mmol) in 100 mL acetonitrile was stirred at refluxing temperature for 24 h. Then after filtration, the solvent was removed in a vacuum. The residue was purified by column chromatography (neutral alumina, dichloromethane/methanol=1/10) to obtain Compound 4 as a brown solid (1.02 g, 43.01% yield). ^{1}H NMR (600 MHz, DMSO-d_6) δ (ppm): 8.51-8.50 (d, *J* = 4.8 Hz, 2H), 7.80-7.77 (m, 2H), 7.51-7.50 (d, *J* = 7.8 Hz, 2H), 7.28-7.26 (m, 2H), 3.81 (s, 4H), 3.36-3.35 (d, *J* = 2.4 Hz, 2H), 3.23-3.22 (t, 1H). ^{13}C NMR (500 MHz, $CDCl_3$) δ (ppm): 158.80, 149.28, 136.50, 123.18, 122.13, 78.38, 73.65, 59.48 and 42.62. HR-MS (ESI) m/z for $C_{15}H_{15}N_3Na$ $[M+Na]^+$ calcd. 260.1158, found: 260.1164.

Synthesis of N, N-bis(pyridin-2-ylmethyl) octa-2,4,7-triyn-1-amine (Cu^2R_1).

A mixture of CuI (0.95 g, 5.00 mmol) and TMEDA (1.50 mL, 10.00 mmol) in 25.00 mL of acetone was stirred at 25 °C for 10 min with the constant bubbling of air. Then, Compound 4 (0.71 g, 3.00 mmol) and trimethyl (penta-1, 4-diyn-1-yl) silane (0.48 g, 3.5 mmol) in 10.00 ml dichloromethane were added to the above solution, and the mixture was stirred at 25 °C with air for 2 h. After the reaction was completed, the mixture was filtered to collect the filtrate, and the solvent was removed in a vacuum. The crude product *N*, *N*-bis(pyridin-2-ylmethyl)-8-(trimethylsilyl) octa-2,4,7-triyn-1-amine was purified by column chromatography (neutral alumina, methanol/dichloromethane=1/10) to give a black viscous oil (0.38 g, 34.14% yield). Then, N, N-

bis(pyridin-2-ylmethyl)-8-(trimethylsilyl) octa-2,4,7-triyn-1-amine (0.38 g, 1.00 mmol) was dissolved in methanol (15 mL) and K_2CO_3 (0.17 g, 1.20 mmol) was added. The reaction mixture was allowed to stir at room temperature for 3 h, then removed K_2CO_3 by filtration. The combined filtrate was evaporated and purified through column chromatography (neutral alumina, dichloromethane/methanol=1/10). Brownish-black solid Cu^2R_1 was collected (0.26 g, 86.67% yield). ^1H NMR (500 MHz, $CDCl_3$) δ (ppm): 8.53-8.52 (d, J = 5.0 Hz, 2H), 7.69-7.66 (t, 2H), 7.63-7.62 (d, J = 8.0 Hz, 2H), 7.17-7.14 (t, 2H), 3.88 (s, 4H), 3.74-3.72 (t, 2H), 3.54 (s, 2H), 2.08-2.07 (t, 1H). ^{13}C NMR (500 MHz, $CDCl_3$) δ (ppm): 159.43, 148.94, 136.49, 122.98, 122.04, 83.19, 71.12, 70.39, 68.55, 67.43, 66.10, 60.09, 42.53 and 9.61. HR-MS (ESI) m/z for $C_{20}H_{17}N_3Na$ $[M+Na]^+$ calcd. 322.1471, found: 322.1465.

2. Supplementary Discussion

Calculation of enhancement factor.

In the experiment, 10 μL of ethanol solution of EETP (0.1 M) was dried onto the silicon wafer (0.4 × 0.4 cm^{-2}) and N_{bulk} can be estimated as:

$N_{bulk} = 10\ \mu L \times 0.1\ mol\ L^{-1} \times 6.02 \times 10^{23}\ mol^{-1} \times 11.53\ \mu m^2/0.16\ cm^{-2}$

where d was the diameter of the light spot $d = 1.22\ \lambda/NA$, λ was the excitation light wavelength of 785 nm, the numerical aperture of the objective lens N. A = 0.25, thereby, Laser spot size ($\pi\ (d/2)^2$) was estimated to about 11.53 μm^2. N_{bulk} was estimated to be 4.34×10^{11}. N_{ads} was determined by laser spot illuminating on the sample and density of EETP molecule adsorbed on the surface of GNs: $N_{ads} = V_{lens}\ C_{GNs}\ NA\ A_{GNs}\ \rho_{ads}\ NA$.

A single particle containing ~ 10 sharp tips branching out from a central spherical core, and a normally distributed branch length of 14.50±4.40 nm was considered with a width of 10.10±2.40 nm at the base and a tip apex diameter of 4.90±1.10 nm, and the core diameter was fixed at 20.50±3.40 nm. Thus, the surface area A_{GNs} of the individual particle was calculated to be ~4744.04 nm^2, and GNs were mixed with an excess of EETP, and resuspended by centrifugation to obtain GNs with the surface maximally modified by EETP (0.5 mg mL^{-1}). N_{ads} was the number of EETP in the excitation volume for GNs colloids. The excitation volume was calculated by using the equation: $V_{lens} = \pi(d/2)^2H$, where d was the diameter of the beam size (d = 3.83 μm), H was the effective depth of focus (H = 22 μm), the effective excitation volume was 253.46 μm^3 for Raman microscopy with 785 nm excitation using the 10× objective. $\rho_{ads,MBN}$ was the bonding density of EETP onto GNs surface as reported (~49.89±9.12 pmol cm^{-2}).[31] We calculated: N_{ads} = ~4.35 $\times 10^5$, I_{bulk} = 123.28, $I_{SERS,\ GNs}$ = 917.27. EF was estimated to be (7.42±0.63) × 10^6.

3. Supplementary Figures.

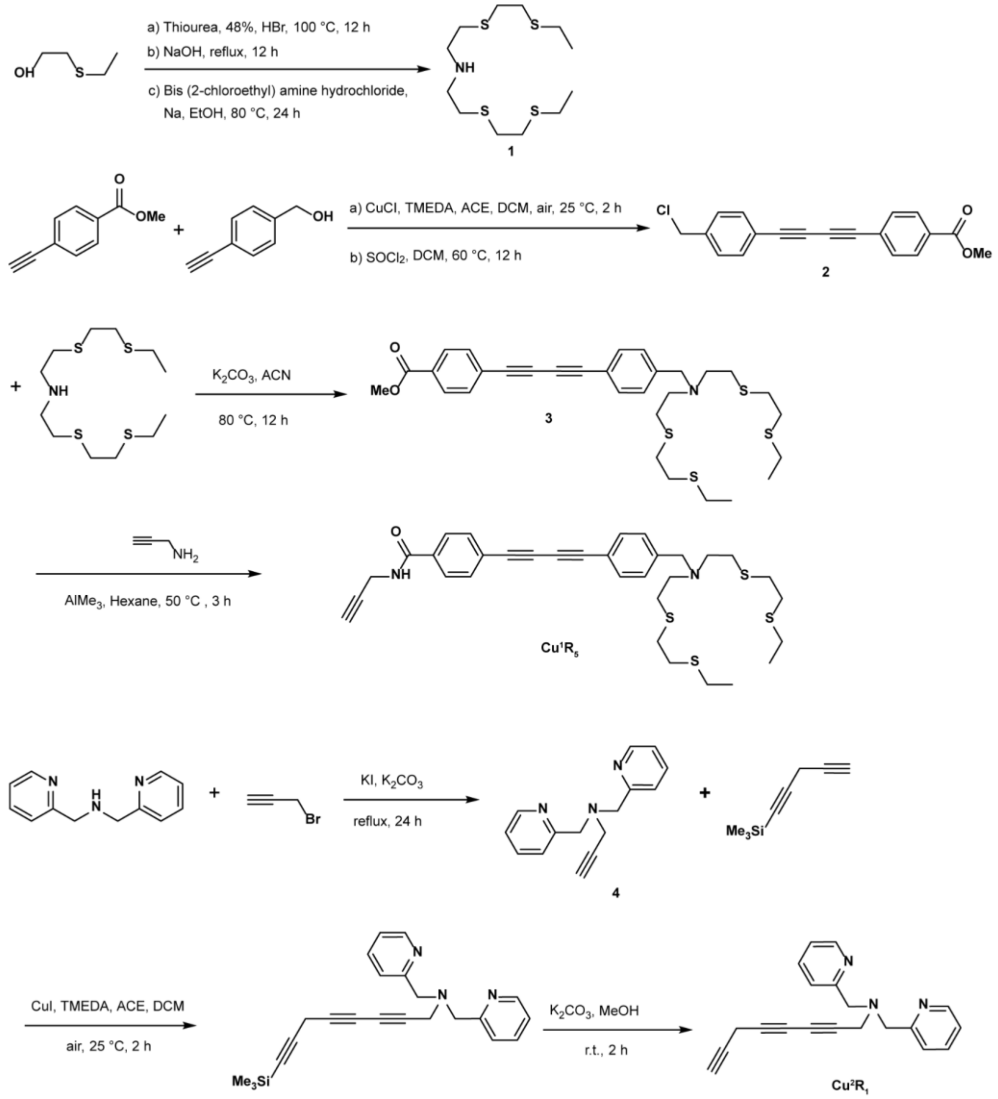

Supplementary Fig. 1 | The synthesis procedures of molecules. The synthesis procedures of probes Cu^1R_5 and Cu^2R_1, and reference compounds

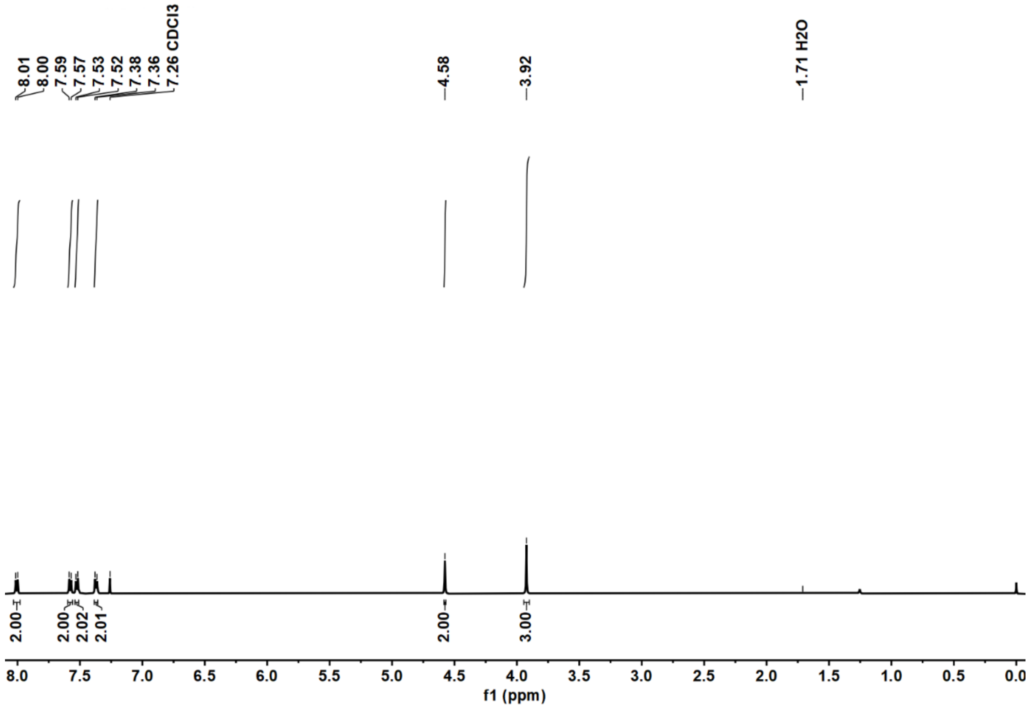

Supplementary Fig. 2 | . ^{1}H NMR of Compound 2 (25 °C).

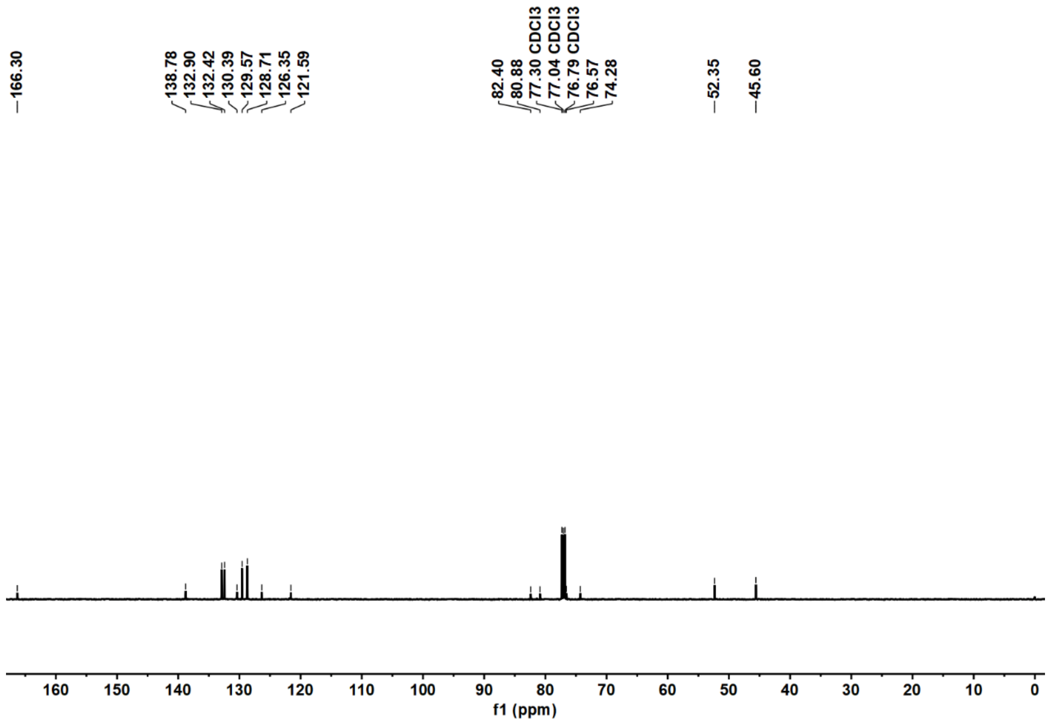

Supplementary Fig. 3 | ^{13}C NMR of Compound 2 (25 °C).

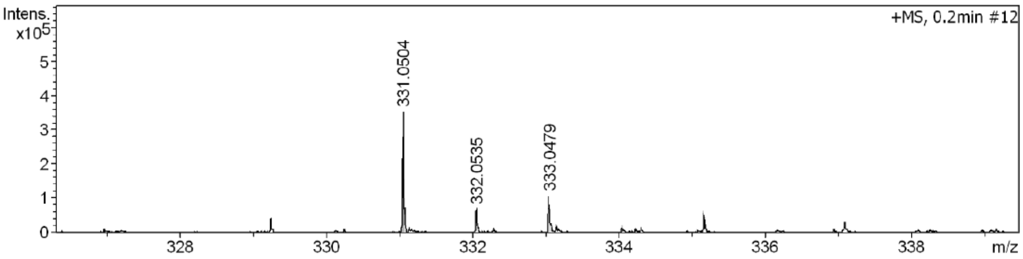

#	m/z	Res.	S/N	I	I %	FWHM
1	331.0504	26996	544.8	351240	100.0	0.0123
2	332.0535	21761	104.5	67464	19.2	0.0153
3	333.0479	20802	164.1	106232	30.2	0.0160

Meas. m/z	#	Ion Formula	m/z	err [ppm]	mSigma		Score	rdb	e⁻ Conf	N-Rule
331.0504	1	C19H13ClNaO2	331.0496	-2.4	20.3	1	100.00	12.5	even	ok

Supplementary Fig. 4 | ESI-HRMS spectrum of Compound 2 (25 °C).

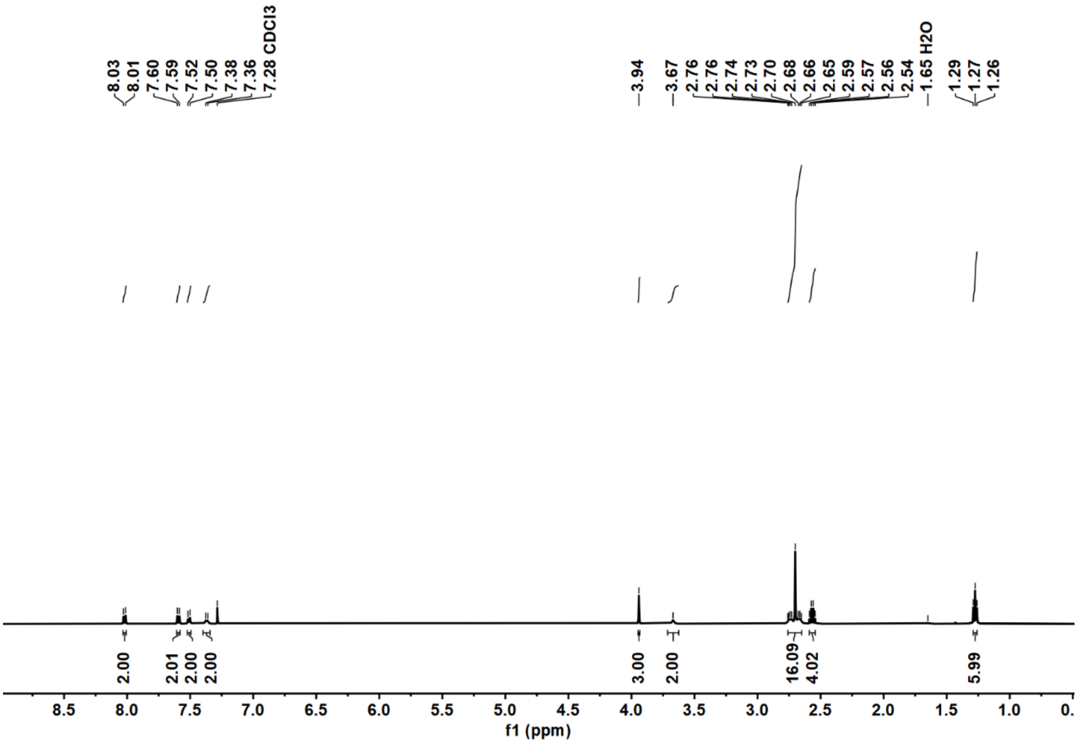

Supplementary Fig. 5 | ^{1}H NMR of Compound 3 (25 °C).

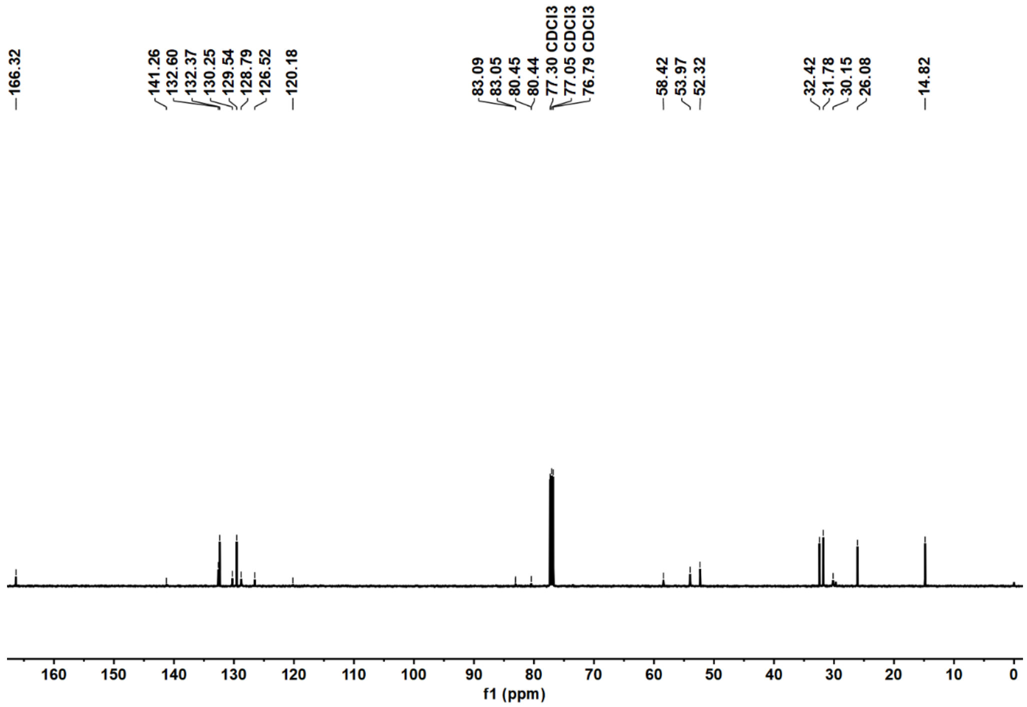

Supplementary Fig. 6 | ^{13}C NMR of Compound 3 (25 °C).

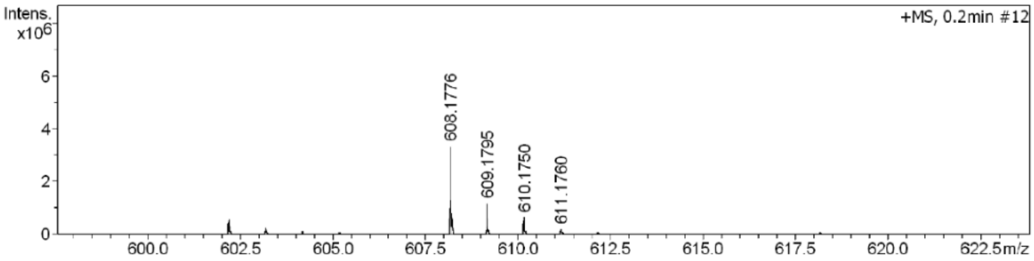

#	m/z	Res.	S/N	I	I %	FWHM
1	608.1776	27047	3751.6	3318644	100.0	0.0225
2	609.1795	38220	1306.6	1153240	34.8	0.0159
3	610.1750	30670	726.5	639644	19.3	0.0199
4	611.1760	24044	207.4	182384	5.5	0.0254

Meas. m/z	#	Ion Formula	m/z	err [ppm]	mSigma	#	Score	rdb	e⁻ Conf	N-Rule
608.1776	1	C31H39NNaO2S4	608.1756	-3.3	28.2	2	69.68	12.5	even	ok

Supplementary Fig. 7 | ESI-HRMS spectrum of Compound 3 (25 °C).

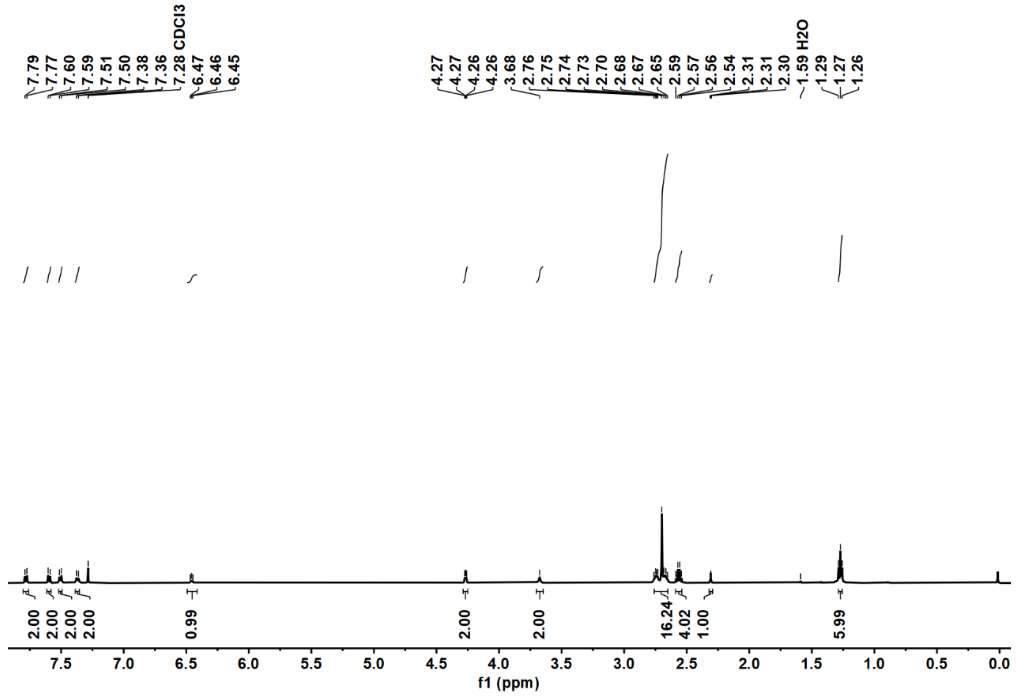

Supplementary Fig. 8 | ^{1}H NMR of $Cu^{1}R_{5}$ (25 °C).

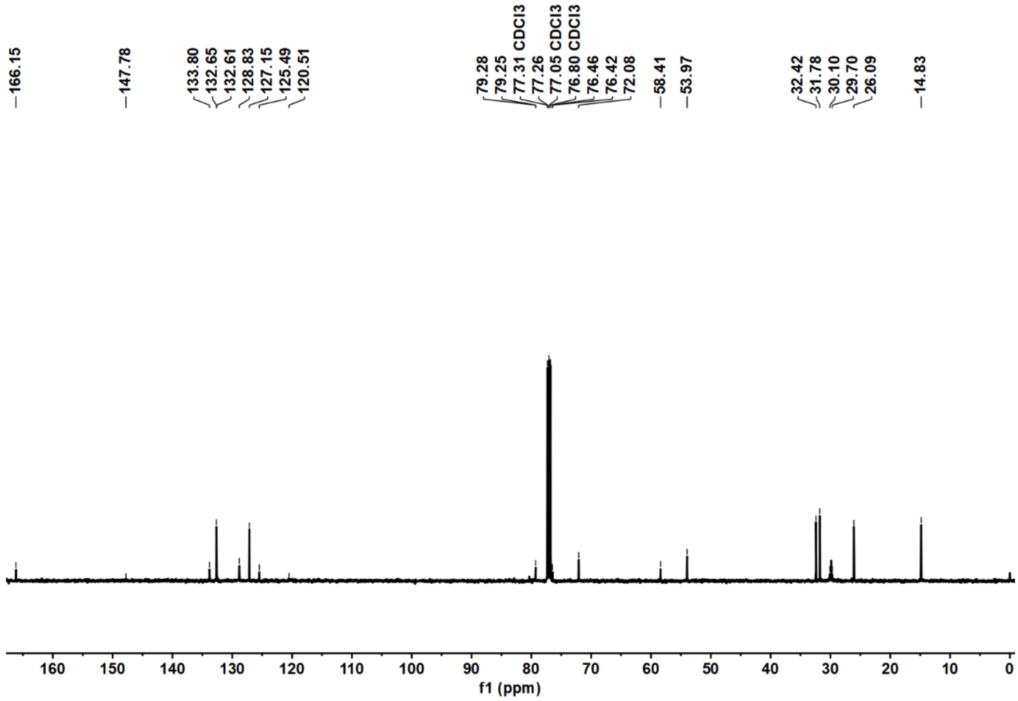

Supplementary Fig. 9 | ^{13}C NMR of $Cu^{1}R_{5}$ (25 °C).

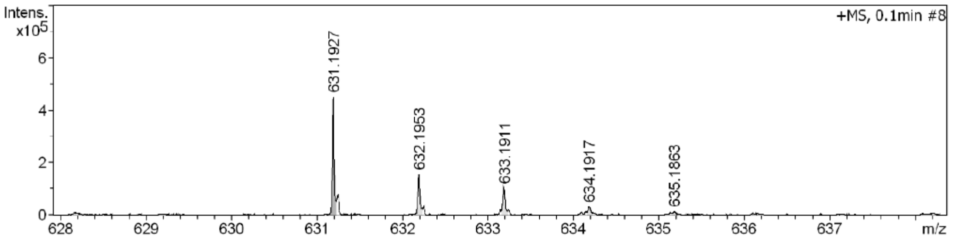

#	m/z	Res.	S/N	I	I %	FWHM
1	631.1927	31374	445.9	448896	100.0	0.0201
2	632.1953	24014	152.5	153216	34.1	0.0263
3	633.1911	20779	100.4	100652	22.4	0.0305
4	634.1917	18290	31.2	31204	7.0	0.0347
5	635.1863	18420	13.8	13792	3.1	0.0345

Meas. m/z	#	Ion Formula	m/z	err [ppm]	mSigma		Score	rdb	e⁻ Conf	N-Rule
631.1927	1	C33H40N2NaOS4	631.1916	-1.7	28.7	5	46.68	14.5	even	ok

Supplementary Fig. 10 | ESI-HRMS spectrum of Cu[1]R$_5$ (25 °C).

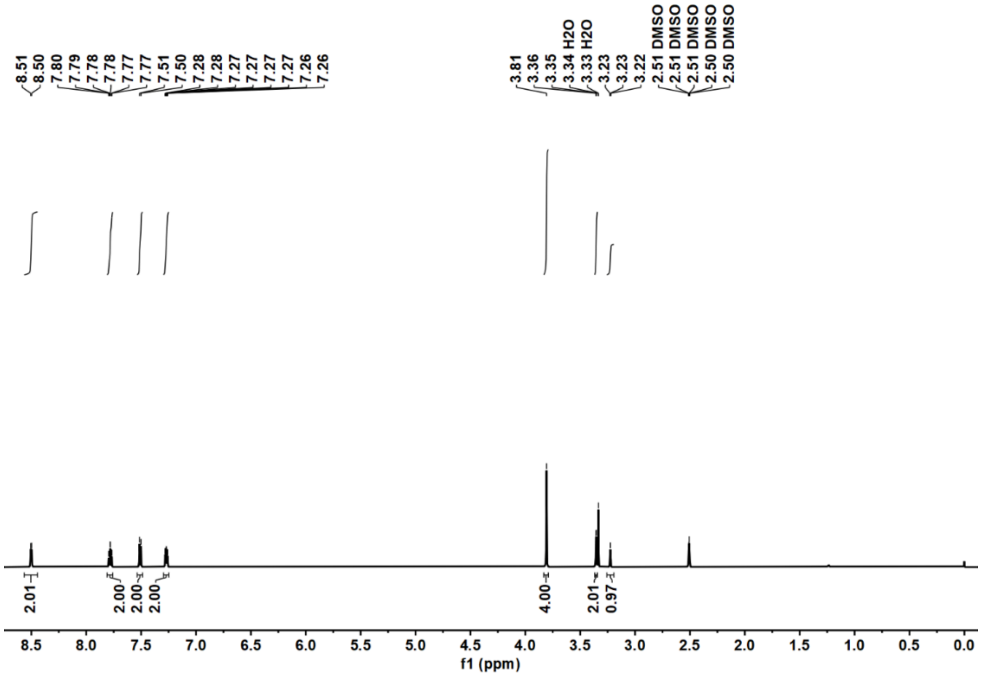

Supplementary Fig. 11 | ^{1}H NMR of Compound 4 (25 °C).

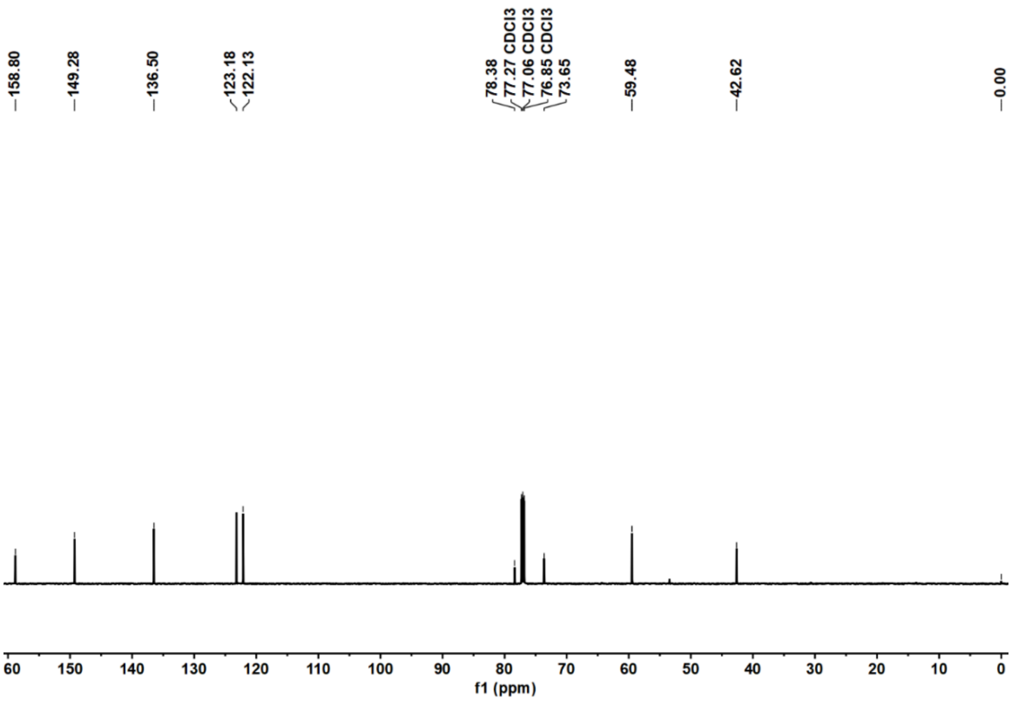

Supplementary Fig. 12 | ^{13}C NMR of Compound 4 (25 °C).

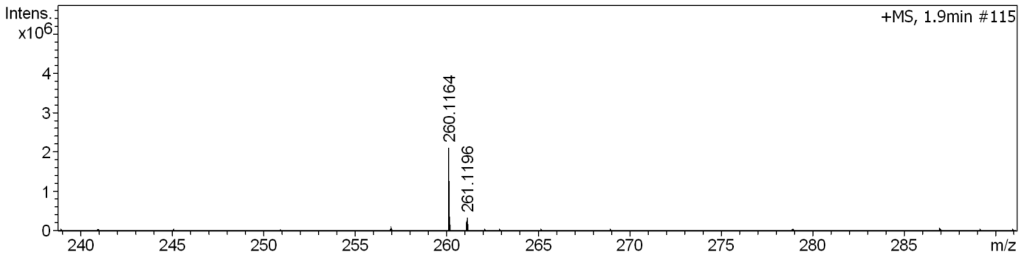

#	m/z	Res.	S/N	I	I %	FWHM
1	260.1164	20446	13348.8	2118312	100.0	0.0127
2	261.1196	14483	2087.6	333056	15.7	0.0180
3	262.1237	10148	185.7	29925	1.4	0.0258

Meas. m/z	#	Ion Formula	m/z	err [ppm]	mSigma		Score	rdb	e⁻ Conf	N-Rule
260.1164	1	C15H15N3Na	260.1158	-2.1	10.2	1	100.00	9.5	even	ok

Supplementary Fig. 13 | ESI-HRMS spectrum of Compound 4 (25 °C).

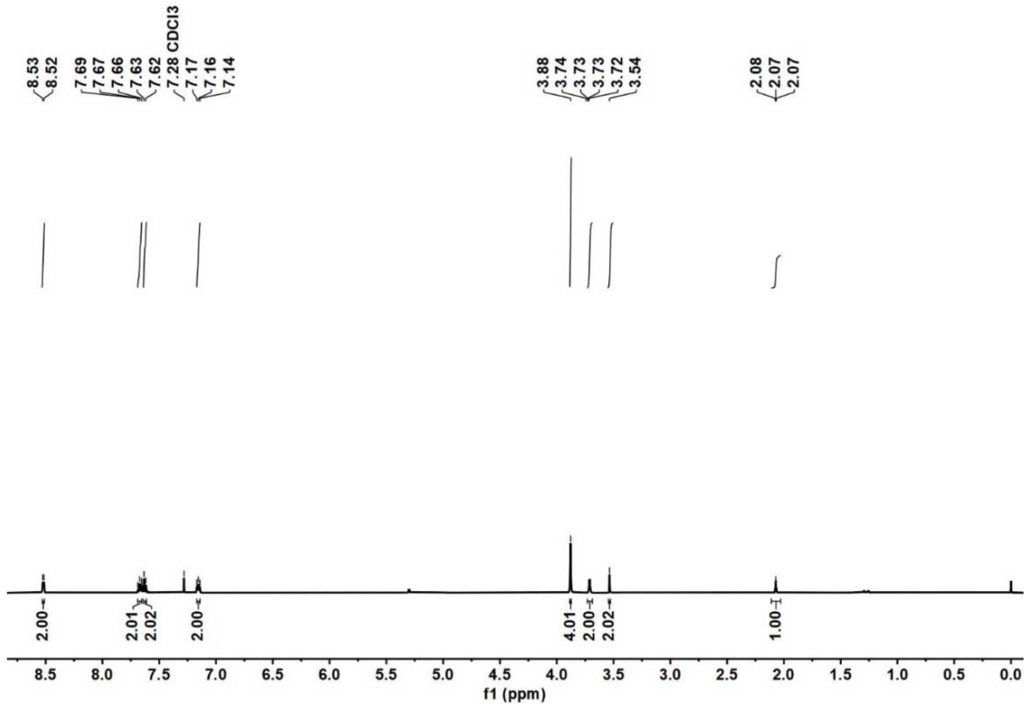

Supplementary Fig. 14 | ^{1}H NMR of Cu^{2}R$_{1}$ (25 °C).

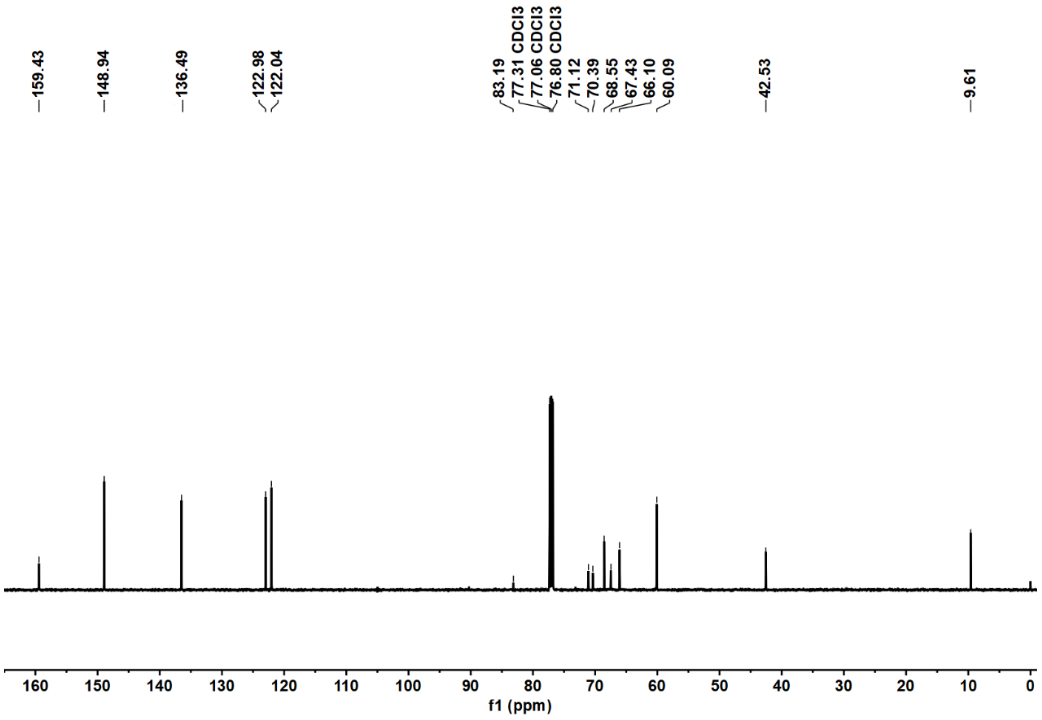

Supplementary Fig. 15 | ^{13}C NMR of Cu^{2}R$_{1}$ (25 °C).

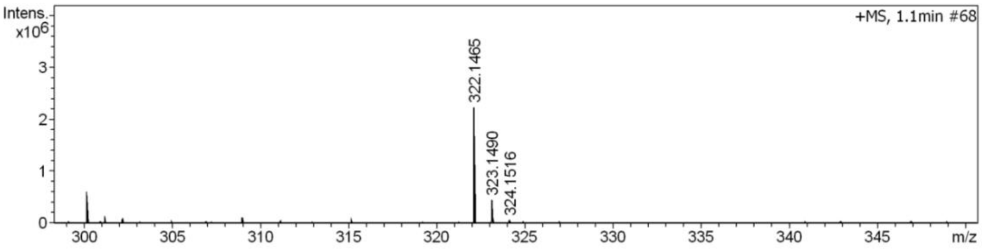

#	m/z	Res.	S/N	I	I %	FWHM
1	322.1465	20117	1754.4	2222660	100.0	0.0155
2	323.1490	18488	350.4	444052	20.0	0.0169
3	324.1516	13452	35.4	44884	2.0	0.0234

Meas. m/z	#	Ion Formula	m/z	err [ppm]	mSigma		Score	rdb	e⁻ Conf	N-Rule
322.1465	1	C20H17N3Na	322.1471	2.0	11.0	1	100.00	11.5	even	ok

Supplementary Fig. 16 | ESI-HRMS spectrum of Cu^2R_1 (25 °C).

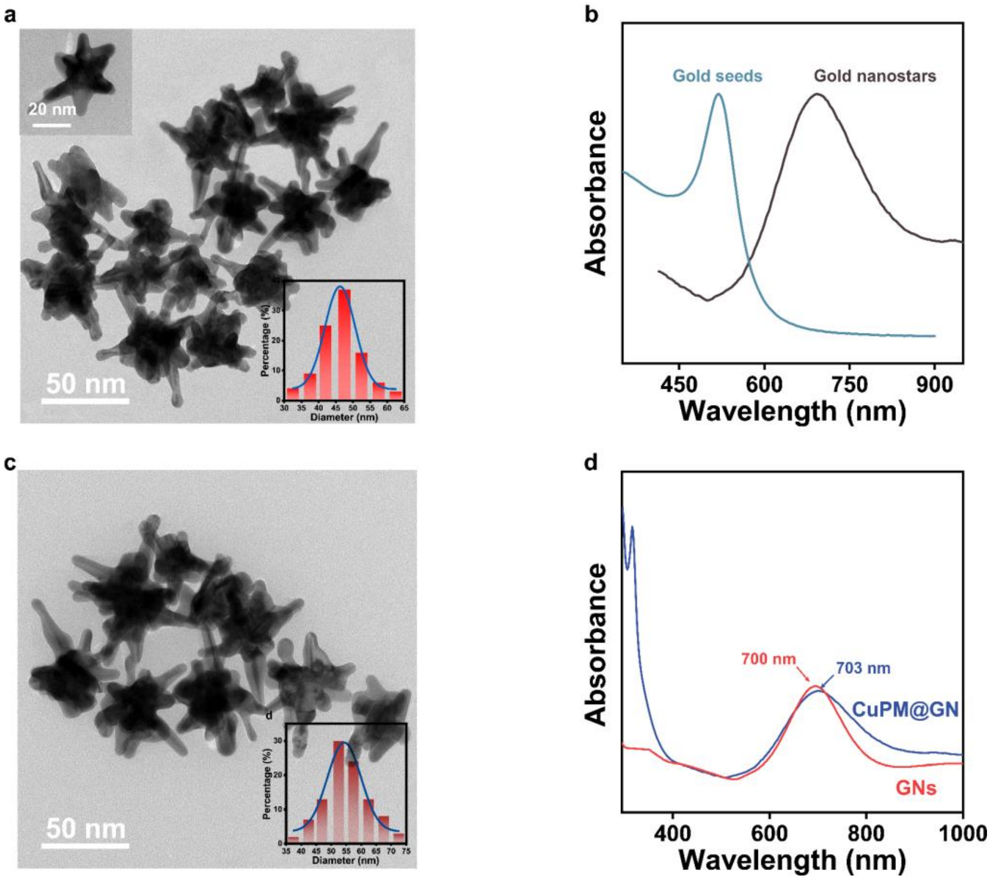

Supplementary Fig. 17 | TEM images and UV-vis spectrums of GNs and CuPM@GN. a TEM images of gold nanostars (GNs). Insets: high-resolution TEM image and diameter distributions of GNs, respectively. **b** UV-vis absorption spectra of gold seeds and GNs. **c** TEM images of CuPM@GN. Insets: diameter distributions. **d** UV-vis spectra of CuPM @GN and GNs, respectively.

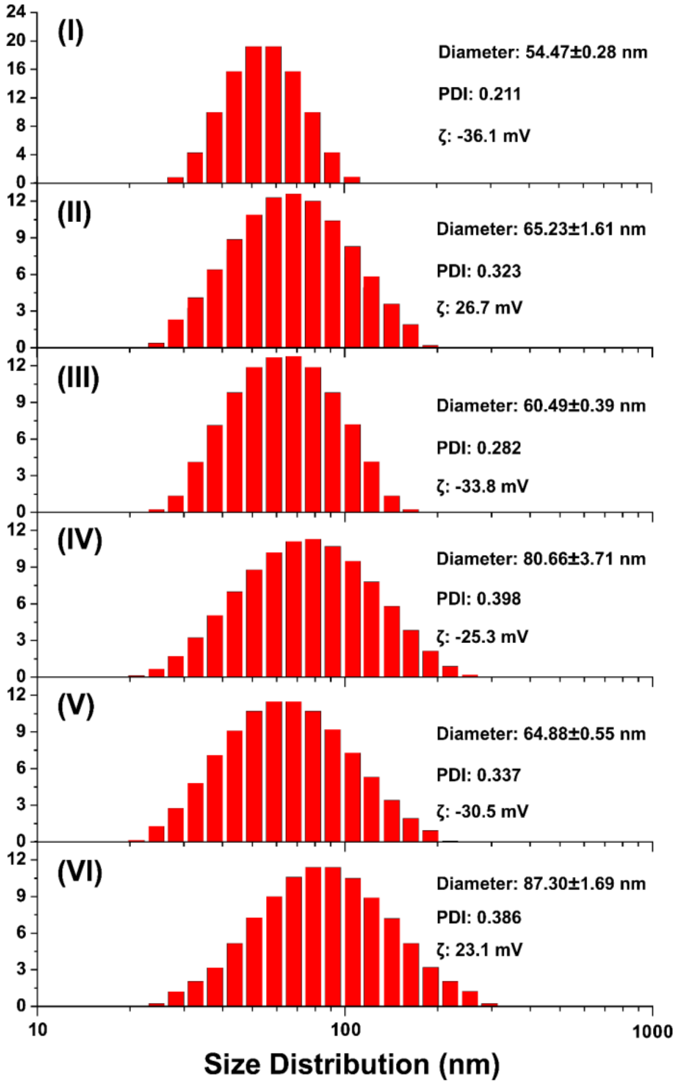

Supplementary Fig. 18 | DLS of various functionalized GNs. Size distribution, polydispersity index (PDI) and zeta potentials (ζ) characterization of (I) GNs, (II) TPP@GN, (III) EETP@GN, (IV) Cu^1R_5@GN, (V) Cu^2R_1@GN and (VI) CuPM@GN, respectively (PBS, 20 mM; pH=7.40).

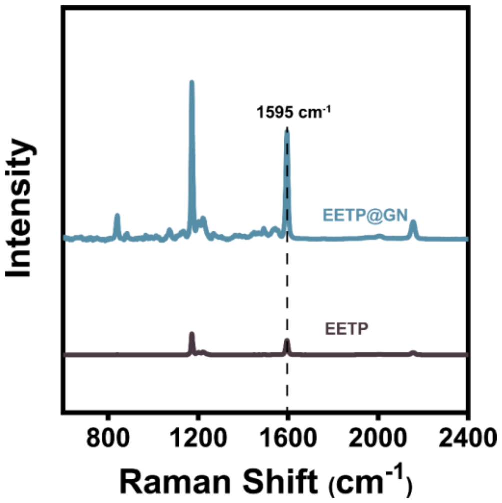

Supplementary Fig. 19 | Enhancement factor. SERS spectrum of EETP on GNs (EETP@GN), and Raman spectrum of an ethanol solution of EETP (0.1 M, 10 μL) dried onto a silicon wafer.

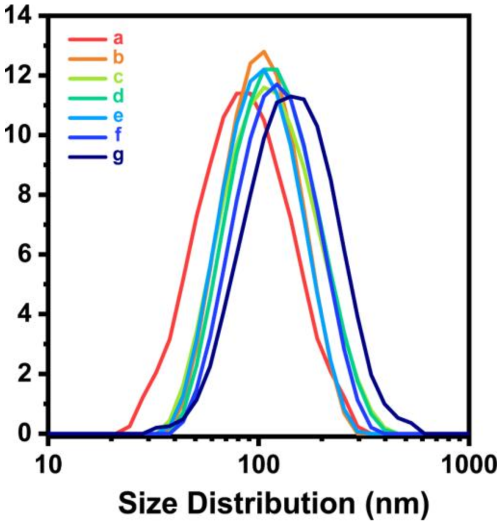

Supplementary Fig. 20 | Hydrodynamic diameters of CuPM@GN upon addition of copper ions. Hydrodynamic diameters of CuPM@GN (0.46 mg mL^{-1}) upon addition of both Cu^{+} (a-g: 1.0, 2.0, 5.0, 10.0, 12.0, 14.0 μM) and Cu^{2+} (a-g: 1.0, 2.0, 5.0, 10.0, 12.0, 16.0 μM) in PBS buffer (20 mM, pH=7.40).

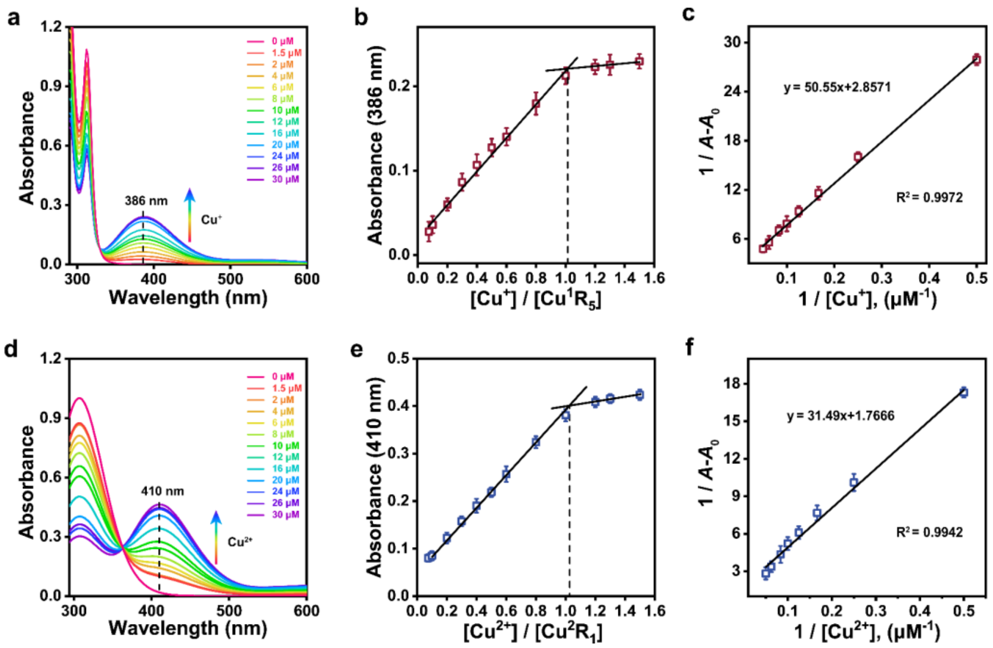

Supplementary Fig. 21 | Binding constants of the Cu^+ to Cu^1R_5 and the Cu^{2+} to Cu^2R_1. a Absorption spectra of Cu^1R_5 (20 μM) upon titration with [Cu ($CH_3CN)_4$][PF_6] from 0 μM (0 eq.) to 30 μM (1.5 eq.) (0, 1.5, 2.0, 4.0, 6.0, 8.0, 10.0, 12.0, 16.0, 20.0, 24.0, 25.0 and 30.0 μM) in a CH_3CN/PBS buffer (1:1, v/v; 20 mM PBS buffer, pH = 7.40) mixture. The peak associated with formation of the complex is at λ_{max} = 386 nm. **b** A plot of absorbance at 386 nm as a function of mole ratio (Cu^+: Cu^1R_5) where the tangents intersect at the metal: complex ratio. Data are presented as mean ± S.D. Error bars represent standard deviation, n = 3 independent replicates. **c** Benesi–Hilderbrand plot of Cu^1R_5 with addition of Cu^+ (2.0-20.0 μM). Data are presented as mean ± S.D. Error bars represent standard deviation, n = 3 independent replicates. **d** Absorption spectra of Cu^2R_1 (20 μM) upon titration with $CuCl_2{\cdot}2H_2O$ from 0 μM (0 eq.) to 30 μM (1.5 eq.) (0, 1.5, 2.0, 4.0, 6.0, 8.0, 10.0, 12.0, 16.0, 20.0, 24.0, 25.0 and 30.0 μM) in a CH_3CN/PBS buffer (1:1, v/v; 20 mM PBS buffer, pH = 7.40) mixture. The peak associated with formation of the complex is at λ_{max} = 410 nm. **e** A plot of absorbance at 410 nm as a function of mole ratio (Cu^{2+}: Cu^2R_1) where the tangents intersect at the metal: complex ratio. Data are presented as mean ± S.D. Error bars represent standard deviation, n = 3 independent replicates. **f** Benesi–Hilderbrand plot of Cu^2R_1 with Cu^{2+} (2.0-20.0 μM). Data are presented as mean ± S.D. Error bars represent standard deviation, n = 3 independent replicates.

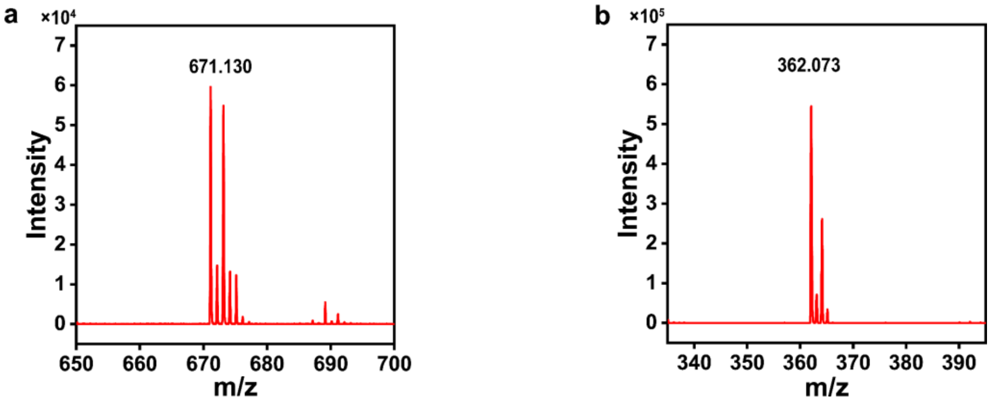

Supplementary Fig. 22 | MALDI spectra of molecule Cu^1R_5 toward Cu^+ and Cu^2R_1 toward Cu^{2+}. a MALDI data of Cu^1R_5 after the addition of Cu^+ (Cu^1R_5-Cu^+). **b** MALDI data of Cu^2R_1 after the addition of Cu^{2+} (Cu^2R_1-Cu^{2+}).

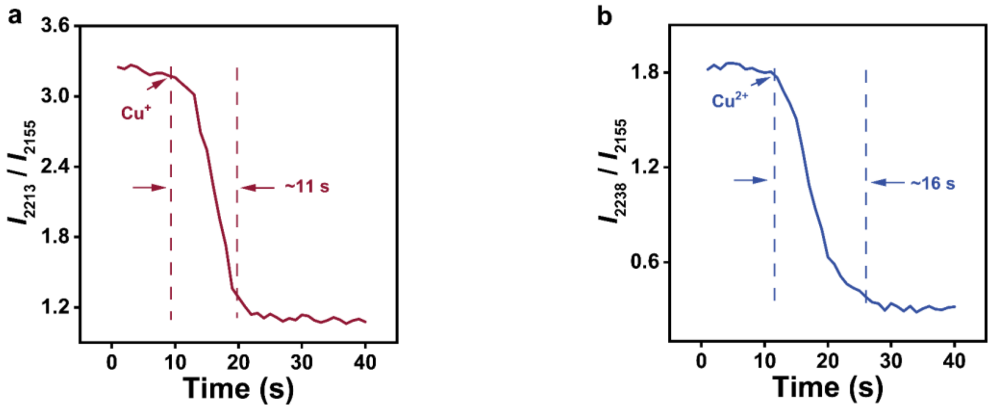

Supplementary Fig. 23 | Response times. a SERS intensity ratio I_{2213}/I_{2155} was obtained from CuPM@GN in PBS (pH=7.4) solution with the addition of 14 μM Cu^+. **b** SERS intensity ratio I_{2238}/I_{2155} was obtained from CuPM@GN in PBS (pH=7.4) solution with the addition of 16 μM Cu^{2+}. The response time was estimated as SERS intensity ratios reached 95% of the maximum values.

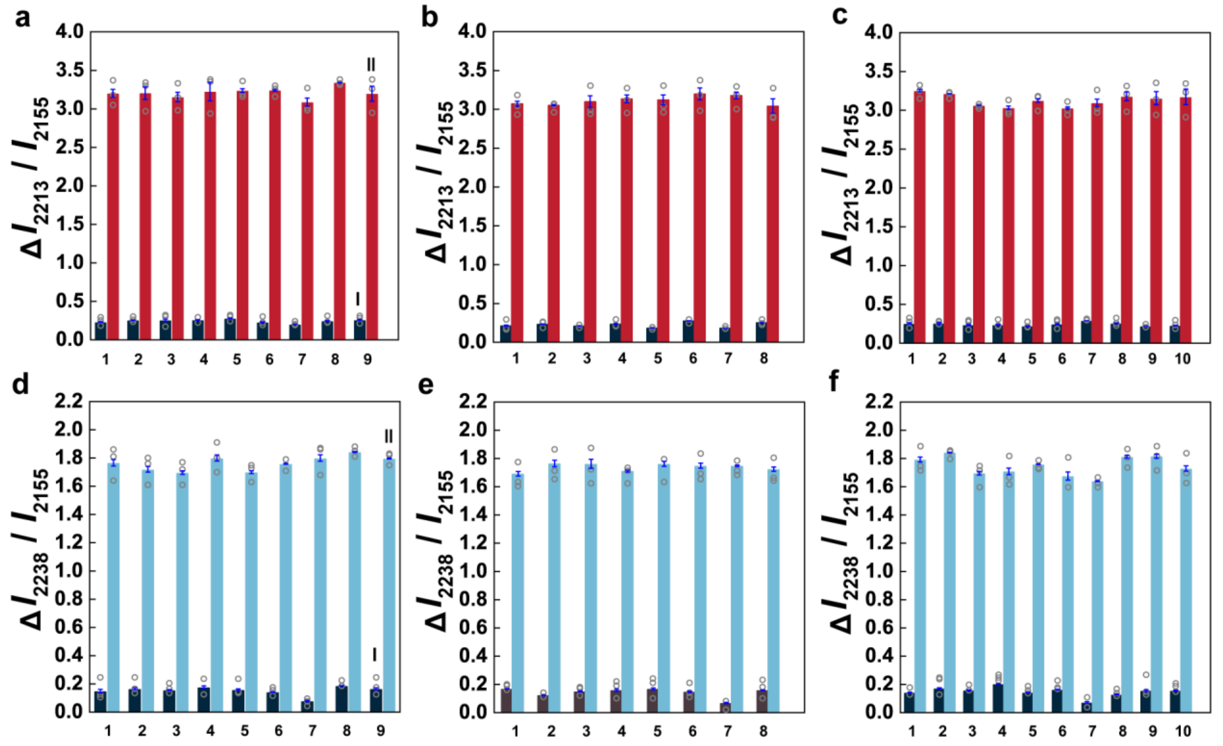

Supplementary Fig. 24 | Selectivity and Competition Tests. a-d Competition (I) and Selectivity (II) tests for Cu^{+} against metal ions (**a**), proteins (**b**) and amino acids (**c**). **a** 1-9: Cu^{2+}, Ca^{2+}, Mg^{2+}, Mn^{2+}, Fe^{2+}, Fe^{3+}, Zn^{2+}, Co^{2+}, Ni^{2+}, 1 mM for Ca^{2+} ,10 μM for Cu^{2+} and 50 μM for other metal ions; **b** 1-8: metallothionein (MT), copper-zinc superoxide dismutase ((Cu, Zn)-SOD), Cox 17, SCO1, Bovine Serum Albumin (BSA), monoamine oxidase A (MAO-A), monoamine oxidase B (MAO-B), catechol-O-methyltransferaseon (COMT), 1.0 mg mL^{-1} BSA, 20 μg mL^{-1} for MT and (Cu, Zn)-SOD, 10 μg mL^{-1} for MAO-A and MAO-B, 5 μg mL^{-1} for Cox 17, SCO1 and COMT. **c** 1-10: Threonine (Thr), Glycine (Gly), Alanine (Ala), Glutamic acid (Glu), Leucine (Leu), Lysine (Lys), Phenylalanine (Phe), Tyrosine (Tyr), GSH, Cysteine (Cys), 500 μM for GSH and 10 μM for all other species. Data are presented as mean ± S.D. Error bars: S.D., $n = 3$ independent experiments. **d-f** Competition (I) and Selectivity (II) tests for Cu^{2+} against metal ions (**d**), proteins (**e**) and amino acids (**f**). **d** 1-9: Cu^{+}, Ca^{2+}, Mg^{2+}, Mn^{2+}, Fe^{2+}, Fe^{3+}, Zn^{2+}, Co^{2+}, Ni^{2+}, 1 mM for Ca^{2+} ,10 μM for Cu^{+} and 50 μM for other metal ions; **e** 1-8: MT, (Cu, Zn)-SOD, Cox 17, SCO1, BSA, MAO-A, MAO-B, COMT, 1.0 mg mL^{-1} BSA, 20 μg mL^{-1} for MT and (Cu, Zn)-SOD, 10 μg mL^{-1} for MAO-A and MAO-B, 5 μg mL^{-1} for Cox 17, SCO1 and COMT. **f** 1-10: Thr, Gly, Ala, Glu, Leu, Lys, Phe, Tyr, GSH, Cys, 500 μM for GSH and 10 μM for all other species. Data are presented as mean ± S.D. Error bars: S.D., $n = 3$ independent experiments. Source data are provided as a Source Data file.

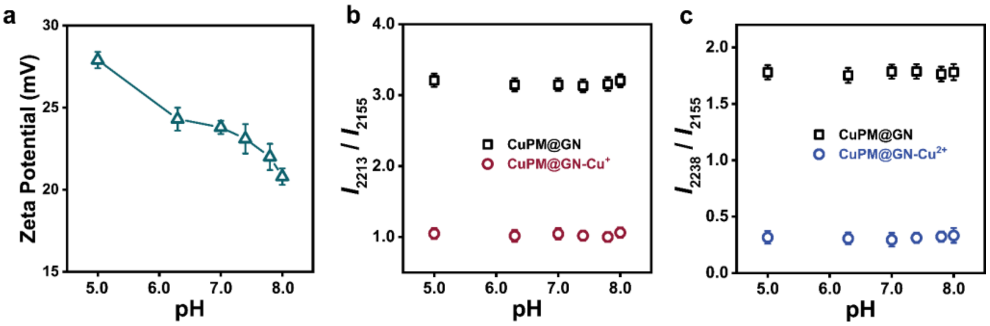

Supplementary Fig. 25 | pH stability. a Zeta potentials of CuPM @GN probe at different pH (5.0, 6.3, 7.0, 7.4, 7.8 and 8.0). **b** SERS intensity ratio I_{2213}/I_{2155} of CuPM@GN without and with the addition of 14 μM Cu^{+} at various pH values from 5.0 to 8.0. **c** SERS intensity ratio I_{2238}/I_{2155} of CuPM@GN without and with the addition of 16 μM Cu^{2+} at various pH values from 5.0 to 8.0. Data are presented as mean ± S.D. Error bars: S.D., n = 3 independent experiments.

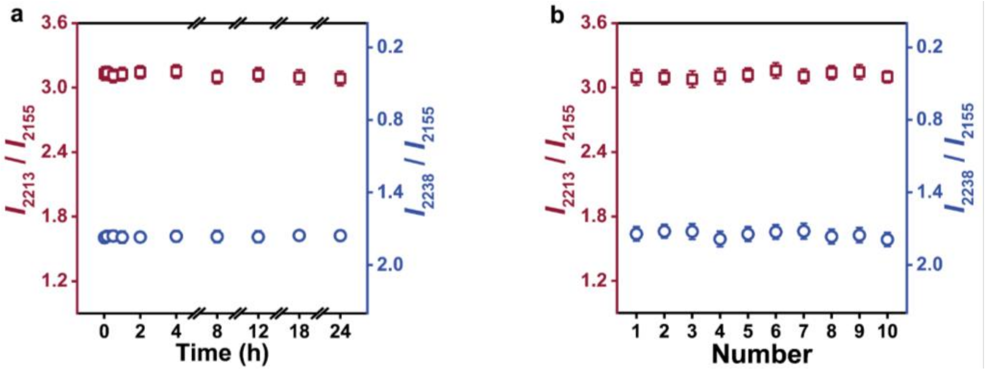

Supplementary Fig. 26 | Time-dependent stability and reproducibility. a Plots of the ratiometric peak intensities (I_{2213}/I_{2155} and I_{2238}/I_{2155}) obtained at different times (1 min, 10 min, 0.5 h, 1 h, 2 h, 4 h, 8 h, 12 h, 18 h, and 24 h). Data are presented as mean ± S.D. Error bars: S.D., n = 3 independent experiments. **b** SERS intensity ratios spectra I_{2213}/I_{2155} and I_{2238}/I_{2155} were acquired from 10 different probes. Data are presented as mean ± S.D. Error bars: S.D., n = 3 independent experiments.

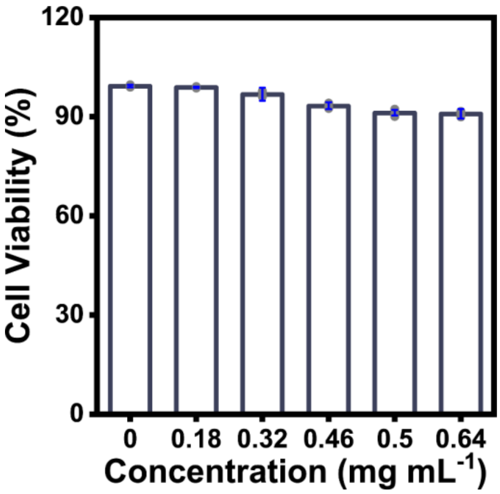

Supplementary Fig. 27 | MTT assay. Cell viability test using MTT assays in neurons incubated with SERS probe CuPM@GN (0, 0.18, 0.32, 0.46, 0.50 and 0.64 mg mL^{-1}) for 24 h. Data are presented as mean ± S.D. Error bars: S.D., n = 3 independent experiments. Source data are provided as a Source Data file.

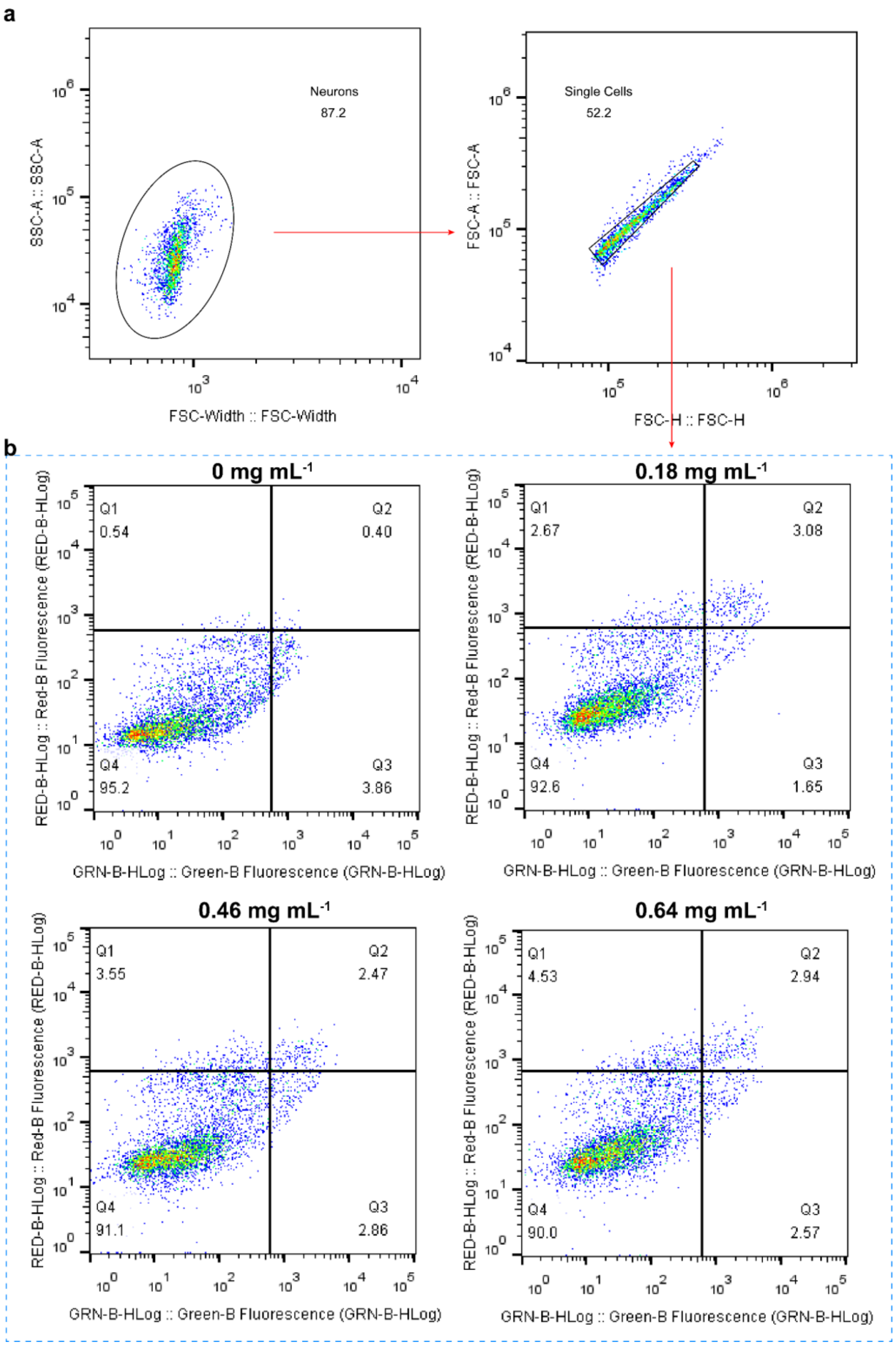

Supplementary Fig. 28 | Apoptosis assay. a Representative FACS gating strategy of neurons sorting. **b** Apoptosis assays of neurons after incubated with CuPM@GN at concentrations of 0, 0.18, 0.46, and 0.64 mg mL^{-1} for 24 h. Q1, Q2, Q3 and Q4 represent the regions of live cells, early apoptotic cells, late apoptotic cells, and dead cells respectively.

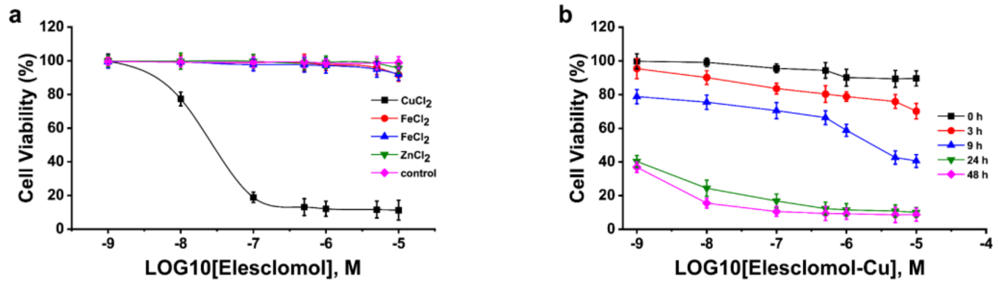

Supplementary Fig. 29 | Copper overload–induced neuron death. a, Viability of cells after treatment with elesclomol (0.001, 0.01, 0.1, 0.5, 1, 5 and 10 μM) with or without 10 μM of indicated metals. Data are presented as mean ± S.D. Error bars: S.D., $n = 3$ independent experiments. **b,** Cell viability of neurons was assessed at the indicated times after ES-Cu (1:1 ratio) (0.001, 0.01, 0.1, 0.5, 1, 5 and 10 μM) pulse treatment for 2 h and then growth in fresh media. Data are presented as mean ± S.D. Error bars: S.D., $n = 3$ independent experiments.

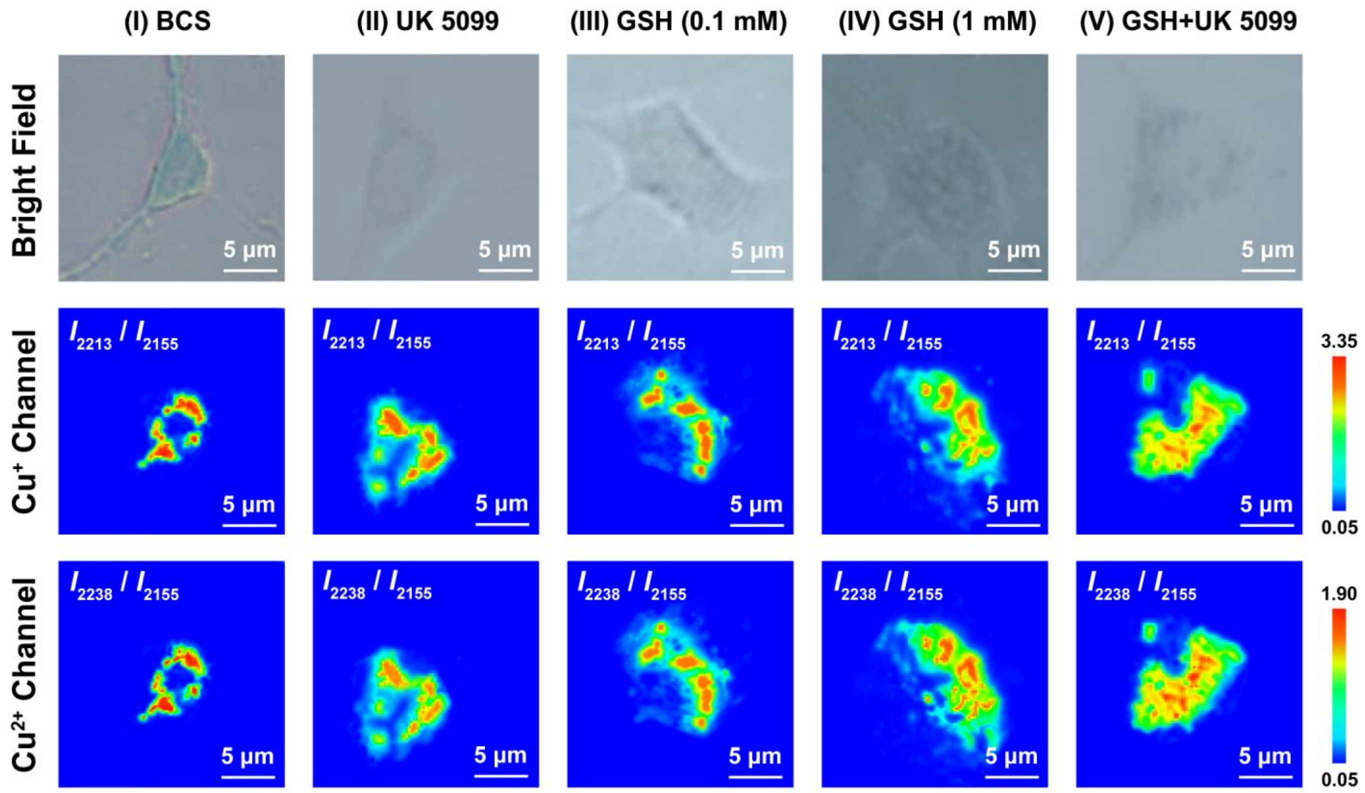

Supplementary Fig. 30 | Imaging of mitochondrial Cu^{+} and Cu^{2+} concentrations. Bright-field images of neurons (top) and SERS imaging of Cu^{+} channel (I_{2213}/I_{2155}, middle) and Cu^{2+} channel (I_{2238}/I_{2155}, bottom) obtained from neurons pre-treated with 20 μM BCS (I), 100 nM UK 5099 (II), 0.1 mM GSH (III), 1 mM GSH (IV), as well as the mixture of 1 mM GSH and 100 nM UK 5099 (V), and then treated with 100 nM ES-Cu for 24 h. Ten independent experiments were repeated and similar results were obtained.

4. Supplementary Tables

Supplementary Table 1. Raman bands assignment of Cu^1R_1 before and after addition of Cu^+.

**Cu^1R_1**		**$Cu^1R_1 + Cu^+$**	
**Shift (cm^{-1})**	**Assignment**	**Shift (cm^{-1})**	**Assignment**
1080	C-C stretching, C-H twisting	1083	C-C stretching, C-H twisting
1121	C-H twisting, C-N stretching	1124	C-H twisting, C-N stretching
1152	C-H twisting	1155	C-H twisting
1212	C-C stretching, C-H twisting	1213	C-C stretching, C-H twisting
1248	C-C stretching, C-H twisting	1250	C-C stretching, C-H twisting
1270	C-H bending	1272	C-H bending
1346	C-C bending , C-H bending	1350	C-C bending , C-H bending
1385	C-H bending	1387	C-H bending
1468	C-H bending	1469	C-H bending
1497	C-H twisting	1499	C-H twisting
2091	HC≡C- stretching	2091	HC≡C- stretching
2236	-C≡C-C≡C- stretching	2238	-C≡C-C≡C- stretching

Supplementary Table 2. Raman bands assignment of Cu^1R_2 before and after addition of Cu^+.

**Cu^1R_2**		**$Cu^1R_2 + Cu^+$**	
**Shift (cm^{-1})**	**Assignment**	**Shift (cm^{-1})**	**Assignment**
1037	Ring breathing	1032	Ring breathing
1120	C-N stretching, C-H twisting	1122	C-N stretching, C-H twisting
1131	C-H bending, C-C stretching	1134	C-H bending, C-C stretching
1203	C-H bending	1207	C-H bending
1235	C-C stretching	1239	C-C stretching
1319	C-H bending	1323	C-H bending
1358	C-H bending	1361	C-H bending
1375	C-C stretching	1380	C-C stretching
1468	C-H bending	1470	C-H bending
1496	C-H bending	1499	C-H bending
1550	C-C stretching, C-H bending	1557	C-C stretching, C-H bending
1606	Ring stretching	1614	Ring stretching
2105	HC≡C- stretching	2105	HC≡C- stretching
2226	-C≡C-C≡C- stretching	2227	-C≡C-C≡C- stretching

Supplementary Table 3. Raman bands assignment of Cu^1R_3 before and after addition of Cu^+.

**Cu^1R_3**		**$Cu^1R_3 + Cu^+$**	
**Shift (cm^{-1})**	**Assignment**	**Shift (cm^{-1})**	**Assignment**
1036	Ring breathing	1029	Ring breathing
1072	C-N stretching, C-C stretching,	1077	C-N stretching, C-C stretching,
1130	C-N stretching, C-H twisting,	1134	C-N stretching, C-H twisting,
1205	C-H bending	1210	C-H bending
1321	C-H twisting	1326	C-H twisting
1362	C-C stretching, C-H bending	1363	C-C stretching, C-H bending
1374	C-H bending	1379	C-H bending
1417	C-H bending, C-C stretching	1419	C-H bending, C-C stretching
1475	C-N stretching, C-H twisting	1477	C-N stretching, C-H twisting
1496	C-H bending	1501	C-H bending
1550	C-C stretching, C-H bending	1557	C-C stretching, C-H bending
1605	Ring stretching	1612	Ring stretching
1673	C=O stretching	1674	C=O stretching
2114	HC≡C- stretching	2114	HC≡C- stretching
2222	-C≡C-C≡C- stretching	2224	-C≡C-C≡C- stretching

Supplementary Table 4. Raman bands assignment of Cu^1R_4 before and after addition of Cu^+.

**Cu^1R_4**		**$Cu^1R_4 + Cu^+$**	
**Shift (cm^{-1})**	**Assignment**	**Shift (cm^{-1})**	**Assignment**
1036	Ring breathing	1029	Ring breathing
1095	C-N stretching, C-C stretching	1098	C-N stretching, C-C stretching
1116	C-N stretching, C-H twisting	1118	C-N stretching, C-H twisting
1133	C-C stretching	1136	C-C stretching
1204	C-H bending	1209	C-H bending
1339	C-C stretching, C-H bending	1345	C-C stretching, C-H bending
1354	C-H bending	1359	C-H bending
1462	C-N stretching, C-H twisting	1467	C-N stretching, C-H twisting
1483	C-H bending	1485	C-H bending
1549	C-C stretching, C-H bending	1554	C-C stretching, C-H bending
1596	Ring stretching	1599	Ring stretching
1687	C=O stretching	1688	C=O stretching
2103	HC≡C- stretching	2103	HC≡C- stretching
2220	-C≡C-C≡C- stretching	2221	-C≡C-C≡C- stretching

Supplementary Table 5. Raman bands assignment of Cu^1R_5 before and after addition of Cu^+.

Cu^1R_5		$Cu^1R_5 + Cu^+$	
**Shift (cm^{-1})**	**Assignment**	**Shift (cm^{-1})**	**Assignment**
1038	Ring breathing	1028	Ring breathing
1114	C-N stretching, C-C stretching	1122	C-N stretching, C-C stretching
1201	C-H bending	1208	C-H bending
1337	C-C stretching, C-H bending	1342	C-C stretching, C-H bending
1349	C-H bending	1353	C-H bending
1442	C-N stretching, C-H twisting	1451	C-N stretching, C-H twisting
1537	C-C stretching, C-H bending	1543	C-C stretching, C-H bending
1595	Ring stretching	1597	Ring stretching
1604	Ring stretching	1613	Ring stretching
1691	C=O stretching	1692	C=O stretching
2121	HC≡C- stretching	2121	HC≡C- stretching
2213	-C≡C-C≡C- stretching	2215	-C≡C-C≡C- stretching

Supplementary Table 6. Raman bands assignment of Cu^2R_1 before and after addition of Cu^{2+}.

Cu^2R_1		$Cu^2R_1 + Cu^{2+}$	
**Shift (cm^{-1})**	**Assignment**	**Shift (cm^{-1})**	**Assignment**
1014	Ring breathing	1008	Ring breathing
1074	C-C stretching, C-H bending	1076	C-C stretching, C-H bending
1160	C-N stretching, C-H twisting	1163	C-N stretching, C-H twisting
1215	C-C stretching, C-H bending	1217	C-C stretching, C-H bending
1238	C-C stretching, C-H bending	1239	C-C stretching, C-H bending
1269	C-C stretching	1271	C-C stretching
1310	C-H bending, C-C bending	1313	C-H bending, C-C bending
1385	C-H bending, C-C bending	1385	C-H bending, C-C bending
1448	C-H bending	1449	C-H bending
1498	C-H bending, C=C stretching	1501	C-H bending, C=C stretching
1617	Ring stretching	1624	Ring stretching
2097	HC≡C- stretching	2097	HC≡C- stretching
2238	-C≡C-C≡C- stretching	2239	-C≡C-C≡C- stretching

Supplementary Table 7. Raman bands assignment of Cu^2R_2 before and after addition of Cu^{2+}.

Cu^2R_2		$Cu^2R_2 + Cu^{2+}$	
**Shift (cm^{-1})**	**Assignment**	**Shift (cm^{-1})**	**Assignment**
1017	Ring breathing	1011	Ring breathing
1038	Ring breathing	1035	Ring breathing
1077	C-C stretching, C-H bending	1078	C-C stretching, C-H bending
1141	C-N stretching, C-H bending	1142	C-N stretching, C-H bending
1167	C-C bending, C-H bending	1169	C-C bending, C-H bending
1198	C-C stretching, C-H twisting	1199	C-C stretching, C-H twisting
1231	C-H twisting	1233	C-H twisting
1323	C-C bending, C-H bending	1325	C-C bending, C-H bending
1375	C-H bending, C-C stretching	1375	C-H bending, C-C stretching
1399	C-H bending, C-C bending	1402	C-H bending, C-C bending
1454	C-H bending	1458	C-H bending
1464	C-H bending	1466	C-H bending
1517	C-H bending, C=C stretching	1519	C-H bending, C=C stretching
1604	Ring stretching	1613	Ring stretching
1614	Ring stretching	1621	Ring stretching
2112	HC≡C- stretching	2112	HC≡C- stretching
2229	-C≡C-C≡C- stretching	2230	-C≡C-C≡C- stretching

Supplementary Table 8. Raman bands assignment of Cu^2R_3 before and after addition of Cu^{2+}.

Cu^2R_3		$Cu^2R_3 + Cu^{2+}$	
**Shift (cm^{-1})**	**Assignment**	**Shift (cm^{-1})**	**Assignment**
1015	Ring breathing	1007	Ring breathing
1036	Ring breathing	1032	Ring breathing
1071	C-C stretching, C-H bending	1072	C-C stretching, C-H bending
1154	C-N stretching, C-H twisting	1154	C-N stretching, C-H twisting
1199	C-H bending	1202	C-H bending
1321	C-C bending, C-H twisting	1325	C-C bending, C-H twisting
1375	C-H bending, C-C stretching	1376	C-H bending, C-C stretching
1416	C-H bending, C-C bending	1417	C-H bending, C-C bending
1429	C-H bending	1433	C-H bending
1476	C-N stretching, C-H twisting	1477	C-N stretching, C-H twisting
1516	C-H bending, C=C stretching	1518	C-H bending, C=C stretching
1603	Ring stretching	1610	Ring stretching
1615	Ring stretching	1622	Ring stretching
1694	C=O stretching	1694	C=O stretching
2127	HC≡C- stretching	2127	HC≡C- stretching
2226	-C≡C-C≡C- stretching	2227	-C≡C-C≡C- stretching

Supplementary Table 9. Raman bands assignment of Cu^2R_4 before and after addition of Cu^{2+}.

**Cu^2R_4**		**$Cu^2R_4 + Cu^{2+}$**	
**Shift (cm^{-1})**	**Assignment**	**Shift (cm^{-1})**	**Assignment**
1016	Ring breathing	1008	Ring breathing
1039	Ring breathing	1036	Ring breathing
1078	C-C stretching, C-H bending	1079	C-C stretching, C-H bending
1151	C-N stretching, C-H twisting	1152	C-N stretching, C-H twisting
1202	C-H bending	1205	C-H bending
1357	C-H bending, C-C stretching	1357	C-H bending, C-C stretching
1402	C-H bending	1405	C-H bending
1411	C-H bending, C-C bending	1412	C-H bending, C-C bending
1464	C-H bending	1465	C-H bending
1481	C-N stretching, C-H bending	1482	C-N stretching, C-H bending
1516	C-H bending, C=C stretching	1519	C-H bending, C=C stretching
1595	Ring stretching	1597	Ring stretching
1614	Ring stretching	1621	Ring stretching
1714	C=O stretching	1715	C=O stretching
2105	HC≡C- stretching	2105	HC≡C- stretching
2224	-C≡C-C≡C- stretching	2226	-C≡C-C≡C- stretching

Supplementary Table 10. Raman bands assignment of Cu^2R_5 before and after addition of Cu^{2+}.

**Cu^2R_5**		**Cu^2R_5 + Cu^{2+}**	
**Shift (cm^{-1})**	**Assignment**	**Shift (cm^{-1})**	**Assignment**
1017	Ring breathing	1008	Ring breathing
1036	Ring breathing	1032	Ring breathing
1077	C-C stretching, C-H bending	1079	C-C stretching, C-H bending
1171	C-N stretching, C-H bending	1172	C-N stretching, C-H bending
1201	C-H bending	1205	C-H bending
1396	C-H bending, C-C stretching	1397	C-H bending, C-C stretching
1402	C-H bending	1404	C-H bending
1412	C-H bending, C-C bending	1414	C-H bending, C-C bending
1435	C-H bending	1438	C-H bending
1481	C-N stretching, C-H bending	1482	C-N stretching, C-H bending
1523	C-H bending, C=C stretching	1526	C-H bending, C=C stretching
1595	Ring stretching	1596	Ring stretching
1604	Ring stretching	1612	Ring stretching
1614	Ring stretching	1623	Ring stretching
1714	C=O stretching	1714	C=O stretching
2125	HC≡C- stretching	2125	HC≡C- stretching
2215	-C≡C-C≡C- stretching	2216	-C≡C-C≡C- stretching

Supplementary Table. 11. SERS bands assignment of CuPM@GN before and after addition of Cu^{+} and Cu^{2+}.

SERS Probes		CuPM@GN + (Cu^{+}, Cu^{2+})	
**Shift (cm^{-1})**	**Assignment**	**Shift (cm^{-1})**	**Assignment**
1027	Ring breathing	1027	Ring breathing
1036	Ring breathing	1028	Ring breathing
1066	C-C stretching, C-H bending	1066	C-C stretching, C-H bending
1161	C-N stretching, C-H twisting	1162	C-N stretching, C-H twisting
1213	C-C stretching, C-H bending	1215	C-C stretching, C-H bending
1269	C-C stretching	1271	C-C stretching
1444	C-H bending	1446	C-H bending
1604	Ring stretching	1613	Ring stretching
1617	Ring stretching	1624	Ring stretching
~2050	-C≡C (: Au) stretching	~2050	-C≡C (: Au) stretching
2155	-C≡C- stretching	2155	-C≡C- stretching
2213	-C≡C-C≡C- stretching	2215	-C≡C-C≡C- stretching
2238	-C≡C-C≡C- stretching	2239	-C≡C-C≡C- stretching

5. Supplementary References

1. Liu, J. et al. Real-time tracking and sensing of Cu^{+} and Cu^{2+} with a single SERS probe in the live brain: toward understanding why copper ions were increased upon ischemia. *Angew. Chem. Int. Ed.* **60**, 21351-21359 (2021).
