# Supplementary material for: Alkyne-tagged SERS nanoprobe for understanding Cu+ and Cu2+ conversion in cuproptosis processes
Source: Nat Commun. 2024 Apr 15;15:3246. doi: 10.1038/s41467-024-47549-1 (PMC11018805; doi:10.1038/s41467-024-47549-1)
Supplement: Supplementary file 3 — Reporting Summary [file 41467_2024_47549_MOESM3_ESM.pdf]

nature portfolio

Corresponding author(s): Yang Tian

Last updated by author(s): Mar 20, 2024

Reporting Summary

Nature Portfolio wishes to improve the reproducibility of the work that we publish. This form provides structure for consistency and transparency in reporting. For further information on Nature Portfolio policies, see our Editorial Policies and the Editorial Policy Checklist.

Statistics

For all statistical analyses, confirm that the following items are present in the figure legend, table legend, main text, or Methods section.

n/a	Confirmed	
☐	☒	The exact sample size (*n*) for each experimental group/condition, given as a discrete number and unit of measurement
☐	☒	A statement on whether measurements were taken from distinct samples or whether the same sample was measured repeatedly
☐	☒	The statistical test(s) used AND whether they are one- or two-sided<br>*Only common tests should be described solely by name; describe more complex techniques in the Methods section.*
☒	☐	A description of all covariates tested
☐	☒	A description of any assumptions or corrections, such as tests of normality and adjustment for multiple comparisons
☐	☒	A full description of the statistical parameters including central tendency (e.g. means) or other basic estimates (e.g. regression coefficient) AND variation (e.g. standard deviation) or associated estimates of uncertainty (e.g. confidence intervals)
☐	☒	For null hypothesis testing, the test statistic (e.g. *F*, *t*, *r*) with confidence intervals, effect sizes, degrees of freedom and *P* value noted<br>*Give P values as exact values whenever suitable.*
☒	☐	For Bayesian analysis, information on the choice of priors and Markov chain Monte Carlo settings
☒	☐	For hierarchical and complex designs, identification of the appropriate level for tests and full reporting of outcomes
☐	☒	Estimates of effect sizes (e.g. Cohen's *d*, Pearson's *r*), indicating how they were calculated

Our web collection on statistics for biologists contains articles on many of the points above.

Software and code

Policy information about availability of computer code

Data collection	The Gaussian 09 suite of programs was used to optimize the configurations. Structural optimization and electrostatic potential surfaces were performed using the b3lyp functional. The basis set used for the C, H, O, N and S atoms was 6-31G (d), whereas the SDD basis set was employed for the Cu atom. The maximum ϕ (ϕmax) and minimum ϕ (ϕmin) of the four materials were obtained with the help of Multiwfn code. The analysis of electrostatic potential (ESP) was performed by Multiwfn 3.4.1, which is a multifunctional wavefunction analysis program. All iso-surface maps were rendered by VMD 1.9.1 program based on the outputs of Multiwfn.
Data analysis	Flow cytometry data was analyzed by Flowlo X10.0.7 R2). Raman data was performed using Renishaw WiRE 5.1 software. Data are presented as mean values±S.D. (standard deviation), calculated using Microsoft Excel 2016. Statistical significance is calculated with an unpaired two-tailed Student's t-test, using IBM SPSS 27 statistical software, followed by post hoc test for multiple comparisons calculated.

For manuscripts utilizing custom algorithms or software that are central to the research but not yet described in published literature, software must be made available to editors and reviewers. We strongly encourage code deposition in a community repository (e.g. GitHub). See the Nature Portfolio guidelines for submitting code & software for further information.

Data

Policy information about availability of data

All manuscripts must include a data availability statement. This statement should provide the following information, where applicable:

- Accession codes, unique identifiers, or web links for publicly available datasets
- A description of any restrictions on data availability
- For clinical datasets or third party data, please ensure that the statement adheres to our policy

All data supporting the findings of this study are available in this paper and the Supplementary Information. Source data are provided with this paper.

Research involving human participants, their data, or biological material

Policy information about studies with human participants or human data. See also policy information about sex, gender (identity/presentation), and sexual orientation and race, ethnicity and racism.

Reporting on sex and gender	All content of this work does not involve human research participants.
Reporting on race, ethnicity, or other socially relevant groupings	All content of this work does not involve human research participants.
Population characteristics	All content of this work does not involve human research participants.
Recruitment	All content of this work does not involve human research participants.
Ethics oversight	All content of this work does not involve human research participants.

Note that full information on the approval of the study protocol must also be provided in the manuscript.

Field-specific reporting

Please select the one below that is the best fit for your research. If you are not sure, read the appropriate sections before making your selection.

☒ Life sciences ☐ Behavioural & social sciences ☐ Ecological, evolutionary & environmental sciences

For a reference copy of the document with all sections, see nature.com/documents/nr-reporting-summary-flat.pdf

Life sciences study design

All studies must disclose on these points even when the disclosure is negative.

Sample size	In general, no calculations were done to determine sample size. n values are also indicated within figure legends which represent biologically independent samples or independent experiments. Biology studies, attempting to have a minimum of n= 3 biological replicates with sufficient reproducibility, especially representative experiments (such as micro graphs). In experiments with western blots, samples size n = 5 correspond to total protein samples obtained from independent cell culture dishes in five independent experiments. For information obtained from a single cell, the number of cells is generally n =10, with similar results.
Data exclusions	No data were excluded from the analyses.
Replication	All experimental findings were replicated at least 3 times with enough reproducibility.
Randomization	The samples were random allocated into experimental groups.
Blinding	The investigators were blinded to group allocation during data collection and/or analysis.

Reporting for specific materials, systems and methods

We require information from authors about some types of materials, experimental systems and methods used in many studies. Here, indicate whether each material, system or method listed is relevant to your study. If you are not sure if a list item applies to your research, read the appropriate section before selecting a response.

Materials & experimental systems

n/a	Involved in the study	
	x	Antibodies
	x	Eukaryotic cell lines
x		Palaeontology and archaeology
	x	Animals and other organisms
x		Clinical data
x		Dual use research of concern
x		Plants

Methods

n/a	Involved in the study	
x		ChIP-seq
	x	Flow cytometry
x		MRI-based neuroimaging

Antibodies

Antibodies used	primary antibody against cytochrome C oxidase 17 (Cox17, 1: 2000, Abcam ab69611); primary antibody against synthesis of cytochrome c oxidase 1 (SCO1, 1:2000, OriGene Technologies, TA381301); primary antibody against dihydrolipoamide S-acetyltransferase (DLAT, 1:1000, OriGene Technologies, TA350579); primary antibody against β-actin (1:5000, Abcam ab8226); Peroxidase-Conjugated Goat Anti-Rabbit (YEASEN 33119ES60)
Validation	Antibody validation has been demonstrated by suppliers and is well documented at respectively website.<br>-Cox17 Rabbit Polyclonal Antibody were previously used (Mol. Nutr. Food Res. 2021, 2065: e2001202. Sci. Rep. 2020, 10: 3571) and further validated in this study (Fig. 4d). Tested applications: ICC/IF, WB, IHC-P. Species reactivity: Human, Mouse, Rat.<br>-SCO1 Rabbit Polyclonal Antibody were previously used and further validated in this study (Fig. 4d). Tested applications: IHC, WB. Species reactivity: Human, Mouse, Rat.<br>-DLAT Rabbit Polyclonal Antibody were previously used and further validated in this study (Fig. 4d). Tested applications: IHC, WB. Species reactivity: Human, Mouse, Rat.

Eukaryotic cell lines

Policy information about cell lines and Sex and Gender in Research

Cell line source(s)	PC-12 (ml096373), SH-SY5Y (ml097536) and HT22 (ml096819) cells were purchased from commercial company (Enzyme-linked Biotechnology Co., Ltd. Shanghai) and the details are available at www.mlbio.cn.
Authentication	PC-12, SH-SY5Y and HT22 cellswere authenticated by the supplier (STR profiling), the details are available at www.mlbio.cn. And we further authenticate each cell line through morphology. PC-12 cells are round and loosely adherent. SH-SY5Y are semi-adherent cells with short tentacle extensions. HT22 cells are neuron-like adherent cells.
Mycoplasma contamination	All cell lines tested negative for mycoplasma contamination.
Commonly misidentified lines (See ICLAC register)	No commonly misidentified cell lines were used.

Animals and other research organisms

Policy information about studies involving animals; ARRIVE guidelines recommended for reporting animal research, and Sex and Gender in Research

Laboratory animals	Newborn within 24 hours C57BL/6 wild-type mice. The housing facility is maintained at 22 ℃ and 35%-55% humidity on a 12-h light/dark cycle (lights on at 8:00 am).
Wild animals	The study did not involve wild animals.
Reporting on sex	The present research did not involve sex-differentiated experiments, and were not applied to only one sex. Due to the small sample size, sex of the mouse was not considered in the study design, and information on sex of mouse was not collected in the study.
Field-collected samples	The study did not involve samples collected from the field.
Ethics oversight	Animal experiments were reviewed and approved by the Animal Care and Use Committee of East China Normal University

Note that full information on the approval of the study protocol must also be provided in the manuscript.

Plants

Seed stocks

All content of this work does not involve plants research participants.

Novel plant genotypes

All content of this work does not involve plants research participants.

Authentication

All content of this work does not involve plants research participants.

Flow Cytometry

Plots

Confirm that:

- [x] The axis labels state the marker and fluorochrome used (e.g. CD4-FITC).
- [x] The axis scales are clearly visible. Include numbers along axes only for bottom left plot of group (a 'group' is an analysis of identical markers).
- [x] All plots are contour plots with outliers or pseudocolor plots.
- [x] A numerical value for number of cells or percentage (with statistics) is provided.

Methodology

Sample preparation

Newborn within 24 hours C57BL/6 wild-type mice were anesthetized with halothane, and then the whole brain tissues were removed quickly and put in Hanks' balanced salt solution (HBSS, free of Mg2+ and Ca2+) in an ice bath, Mouse cortical tissues were quickly striped and cultured in papain for 15 min at 37 ℃, after that they were dispersed into poly-d-lysine-coated 35 mm Petri dishes at a density of 1 x 10^6 cells/dish, Neurons were cultured with neurobasal medium containing L-Glutamineand B27 (37 ℃, 5% C02, 95% 02) and the medium was changed three times a week. Different concentrations of nanoprobe were cultured with neurons for 24 h. After removing the culture media, the cells were collected with the help of EDTA-free trypsin. After washing with PBS, the cells were re-suspended in 300 µL binding buffer and incubated with 5 µL FITC-Annexin V and 5 µL propidium iodide solution for 30 min in dark. Apoptosis assay was detected at an excitation wavelength of 480 nm.

Instrument

FACS Calibur flow cytometry (Becton, Dickinson and Company, USA)

Software

CellQuest Pro was used for collection and CFCS was used for analysis the flow cytometry data.

Cell population abundance

Cell sorting not employed

Gating strategy

Using the FSC/SSC gating, debris was removed by gating on the main cell population. Positivity threshold for each cell line was defined on the basis of PI positive/Annexin V-FITC negative or PI negative/Annexin V-FITC positive sample. Identical positivity threshold was applied to all samples within cell line.

- [x] Tick this box to confirm that a figure exemplifying the gating strategy is provided in the Supplementary Information.
